# Placental growth factor mediates pathological uterine angiogenesis by activating the NFAT5-SGK1 signaling axis in the endometrium: implications for preeclampsia development

**DOI:** 10.1186/s40659-024-00526-w

**Published:** 2024-08-17

**Authors:** Janet P. Raja Xavier, Toshiyuki Okumura, Melina Apweiler, Nirzari A. Chacko, Yogesh Singh, Sara Y Brucker, Satoru Takeda, Florian Lang, Madhuri S Salker

**Affiliations:** 1 Department of Women’s Health, University of Tübingen, 72076 Calwerstraße 7/6, Tübingen, Germany; 2 Department of Obstetrics and Gynaecology, Juntendo University School of Medicine, Tokyo, Japan; 3 Institute of Medical Genetics and Applied Genomics, University of Tübingen, Tübingen, Germany; 4 Department of Physiology, University of Tübingen, Tübingen, Germany

**Keywords:** PlGF, Endometrium, Placentation, Pregnancy, Preeclampsia, SGK1

## Abstract

After menstruation the uterine spiral arteries are repaired through angiogenesis. This process is tightly regulated by the paracrine communication between endometrial stromal cells (EnSCs) and endothelial cells. Any molecular aberration in these processes can lead to complications in pregnancy including miscarriage or preeclampsia (PE). Placental growth factor (PlGF) is a known contributing factor for pathological angiogenesis but the mechanisms remain poorly understood. In this study, we investigated whether PlGF contributes to pathological uterine angiogenesis by disrupting EnSCs and endothelial paracrine communication. We observed that PlGF mediates a *tonicity-independent* activation of nuclear factor of activated T cells 5 (NFAT5) in EnSCs. NFAT5 activated downstream targets including SGK1, HIF-1α and VEGF-A. In depth characterization of PlGF - conditioned medium (CM) from EnSCs using mass spectrometry and ELISA methods revealed low VEGF-A and an abundance of extracellular matrix organization associated proteins. Secreted factors in PlGF-CM impeded normal angiogenic cues in endothelial cells (HUVECs) by downregulating Notch-VEGF signaling. Interestingly, PlGF-CM failed to support human placental (BeWo) cell invasion through HUVEC monolayer. Inhibition of SGK1 in EnSCs improved angiogenic effects in HUVECs and promoted BeWo invasion, revealing SGK1 as a key intermediate player modulating PlGF mediated anti-angiogenic signaling. Taken together, perturbed PlGF-NFAT5-SGK1 signaling in the endometrium can contribute to pathological uterine angiogenesis by negatively regulating EnSCs-endothelial crosstalk resulting in poor quality vessels in the uterine microenvironment. Taken together the signaling may impact on normal trophoblast invasion and thus placentation and, may be associated with an increased risk of complications such as PE.

**Supplementary Information:**

The online version contains supplementary material available at 10.1186/s40659-024-00526-w.

## Introduction

Pregnancy-associated vascular transformations of the decidua are coordinated by complex cellular mechanisms to induce remodelling at the maternal-fetal interface, which are critical for a healthy pregnancy outcome [1, 2]. Angiogenesis is the development of new vessels from existing blood vessels. In the adult, (healthy) angiogenesis rarely occurs except during wound healing and during repair of the vascular bed after menstruation. Uterine angiogenesis within the decidua is coordinated by several factors secreted from stromal cells, surrounding the endometrial vessels [3, 4]. Any aberrations in remodelling of the uterine vasculature during early pregnancy results in miscarriage or pregnancy disorders such as preeclampsia (PE), fetal growth restriction (FGR) and intrauterine deaths (IUD) or stillbirths [5–7]. PE, a pregnancy-specific pathology is associated with hypertension and multiorgan dysfunction [8]. PE is reported to occur in 5 to 7% of all pregnancies globally, unfortunately this number is rising [9]. In 2022, pregnancies affected by PE were responsible for over 70,000 maternal deaths and 500,000 fetal deaths worldwide [9]. Insufficient vascularization within the uterine decidua, followed by poor placentation at the maternal-fetal interface contributes to ischemia, uteroplacental hypoxia, inflammation and elevated levels of oxidative stress [5, 10, 11]. Women diagnosed with a pre-eclamptic pregnancy are reported to be associated with 4-fold increased risk in future incident heart failure and a 2-fold increase in coronary heart diseases [12, 13]. Currently, there are no tests or treatments to predict the onset of preeclampsia or prevent it. Hence, there exists an urgent unmet clinical need to identify new molecular targets for early diagnosis and therapeutics in PE. Recent studies support that the pathophysiology of PE likely involves endometrial determinants in its pathogenesis [14–18]. Therefore, studying maternal uterine health prior to pregnancy is of importance to identify new molecular pathways driving adverse pregnancy outcomes such as PE.

After menstruation, one of the critical processes of the regenerating endometrium is the regrowth of blood vessels (angiogenesis) [19]. During each menstrual cycle and in early pregnancy, the uterine endothelium becomes activated and undergoes sprouting angiogenesis to increase the size and number of blood vessels in the endometrium [20]. The demand for angiogenic stimulus varies both spatially and temporally across the different menstrual phases [21, 22]. Endometrial stromal fibroblasts are known to secrete biochemical cues (growth factors and cytokines) to induce a pro-angiogenic response in endothelial cells of the spiral arterioles [19, 23, 24]. Employing a 3 dimensional (3D) bioengineered vascularized endometrium on-a-chip model, Ahn et al. highlighted the importance of endometrial stromal cells in stimulating angiogenesis in endothelial cells [23]. Stromal cells exhibit functionally directed proangiogenic cues in regulating microvascular network formation through neo-vessel sprouting of the endothelial cells [23, 25]. From a maternal standpoint, pregnancy is an example of extraordinary rapid histogenesis that is unrivalled in healthy adult tissues. This emphasizes the importance of signaling factors produced by the ‘master’ stromal cells within the uterine environment to influence dynamic angiogenesis processes within the endothelial compartment of spiral arteries. Hence, identifying molecular factors that deregulate the vascularized endometrial microenvironment prior to pregnancy and during early placentation is of critical importance.

Placental growth factor (PlGF) is found in the endometrial stroma and abnormal production of endometrial PlGF may result in pregnancy complications, though the mechanism is yet to be determined [26, 27]. The functional role of PlGF in various biological processes continues to expand, in particular its activity in disease progression [28, 29]. The interplay of PlGF as a pleiotropic cytokine is reported to augment ischemia, hypoxia, inflammatory or malignant processes [30–34]. PlGF shares a biochemical and functional relationship with vascular endothelial growth factor (VEGF-A), that is translated into high synergic activity in physiological and pathological angiogenesis [35, 36]. PlGF-VEGFR1 signaling is reported to modulate angiogenesis and tumour growth by regulating the Dll4-Notch pathway [34]. Inhibition of PlGF is further reported to selectively inhibit pathological angiogenesis [32, 37]. Soluble fms-like tyrosine kinase-1 (sFlt-1), is a circulating anti-angiogenic protein that acts by binding to the receptor binding domains of PlGF and to VEGF thereby preventing its interactions with endothelial receptors [38]. Circulating VEGF concentrations are low through pregnancy whilst free PlGF increases in normal pregnancies [38]. Therefore, free PlGF *a priori* is pivotal for maintaining vascular endothelial cell homeostasis [39].

PlGF is produced in many organs and cells including the human endometrium, decidua, placenta, uterine natural killer cells and trophoblasts cells [40]. Endometrial PlGF is higher in the proliferative phase with expression levels declining in the secretory phase, higher levels of PlGF were associated with implantation failure after IVF [41, 42]. Moreover, gene expression studies of the first trimester decidua prior to the onset of PE compared with healthy pregnancies reveals that local decidual PlGF levels are higher in the PE group [43]. Therefore, it is crucial to identify the underlying local factors that potentially contribute to the clinical manifestation of PE within the decidua. To the best of our knowledge, the potential role of endometrial PlGF- associated physiological vessel development in the endometrium and its relation to pregnancy complications has not been investigated.

Nuclear factor of activated T cells (NFAT5) is part of the Rel family of transcriptional activators [44, 45]. It was originally characterized as a cell volume – regulated transcriptional factor activated by osmotic cell stress [44]. In addition to its well-known osmoprotective role, NFAT5 activation can be mediated independent from tonicity, thus having wider consequences on physiological functions such as development, immune function and cellular stress responses [46–51]. In a recent study, it was revealed that the transcriptional activity of NFAT5 mediates production of angiogenic factors causing neovascularization and angiogenesis associated oedema [52]. In retinal pigment epithelial cells aberrant PlGF signaling *via* NFAT5 activity causes abnormal vessel development in diabetic retinopathy [52]. NFAT5-inducible genes include serum glucocorticoid regulated kinase 1 (SGK1) [51, 53], which is a known activator of hypoxia inducible factor 1 subunit alpha (HIF-1α) proteins and subsequent VEGF-A formation and angiogenesis [54–58]. Thus, these findings so far point to a compelling potential role of PlGF-NFAT5-SGK1 in vessel remodelling and thus warrant further investigation in delineating this signaling axis and its role in endometrial angiogenesis.

In the present study, we studied the effect of PlGF mediated NFAT5 regulation in the endometrial stromal cells (EnSCs). Further, we identified a signaling downstream pathway involving PlGF-NFAT5-SGK1 activation in EnSCs. We also characterized the angiogenic factors secreted by the stromal cells upon PlGF mediated NFAT5 activation. To mimic the effect of secreted angiogenic cues on vessel formation ability we also demonstrated its responsiveness in endothelial (HUVECs) cells. Aberrant PlGF mediated secreted factors impaired trophoblast invasion through the HUVEC monolayer. Furthermore, angiogenic behaviour and trophoblast invasion were reversed by inhibiting SGK1.

Our study reveals that PlGF mediated NFAT5-SGK1 activation in endometrial stromal cells negatively regulate secretion of pro-angiogenic factors within the uterine microenvironment. Additionally, we show that the secreted angiogenic factors activate pathological pathway in endothelial cells resulting in impaired angiogenesis. In summary, aberrant endometrial PlGF expression could lead to dysregulated stromal-endothelial communication leading to poor trophoblast invasion.

## Materials and methods

### Cell culture

Primary human EnSCs (#T0533, Applied Biological Materials Inc) were cultured at 37 °C in a humidified 5% CO_2_ atmosphere in DMEM/F-12 medium (#11039-021, Invitrogen) containing 10% (v/v) dextran coated charcoal stripped (#C6241, Sigma-Aldrich) fetal bovine serum (#10270-106, Invitrogen), 1% (v/v) antibiotic-antimycotic solution (#15240-062, Invitrogen) and 1% (v/v) L-glutamine (#25030-024, Invitrogen). Human umbilical vein endothelial cells (HUVECs) (#C-12,203, Sigma-Aldrich) and GFP-tagged HUVECs (#P20201, Innoprot) were cultured in endothelial growth medium (#C-22,010, PromoCell) with 1% (v/v) antibiotic-antimycotic solution (#15240-062, Invitrogen). Human trophoblast cell line, BeWo cells (#86,082,803, Sigma-Aldrich) were cultured in DMEM/F-12 medium (#11039-021, Invitrogen) containing 10% (v/v) fetal bovine serum (#10270-106, Invitrogen). All work was carried out in a Class I laminar flow hood. All cells were routinely tested for mycoplasma (every 3 months) and always gave a negative result.

### Treatment and transfection of EnSCs

**Fig. 1:**
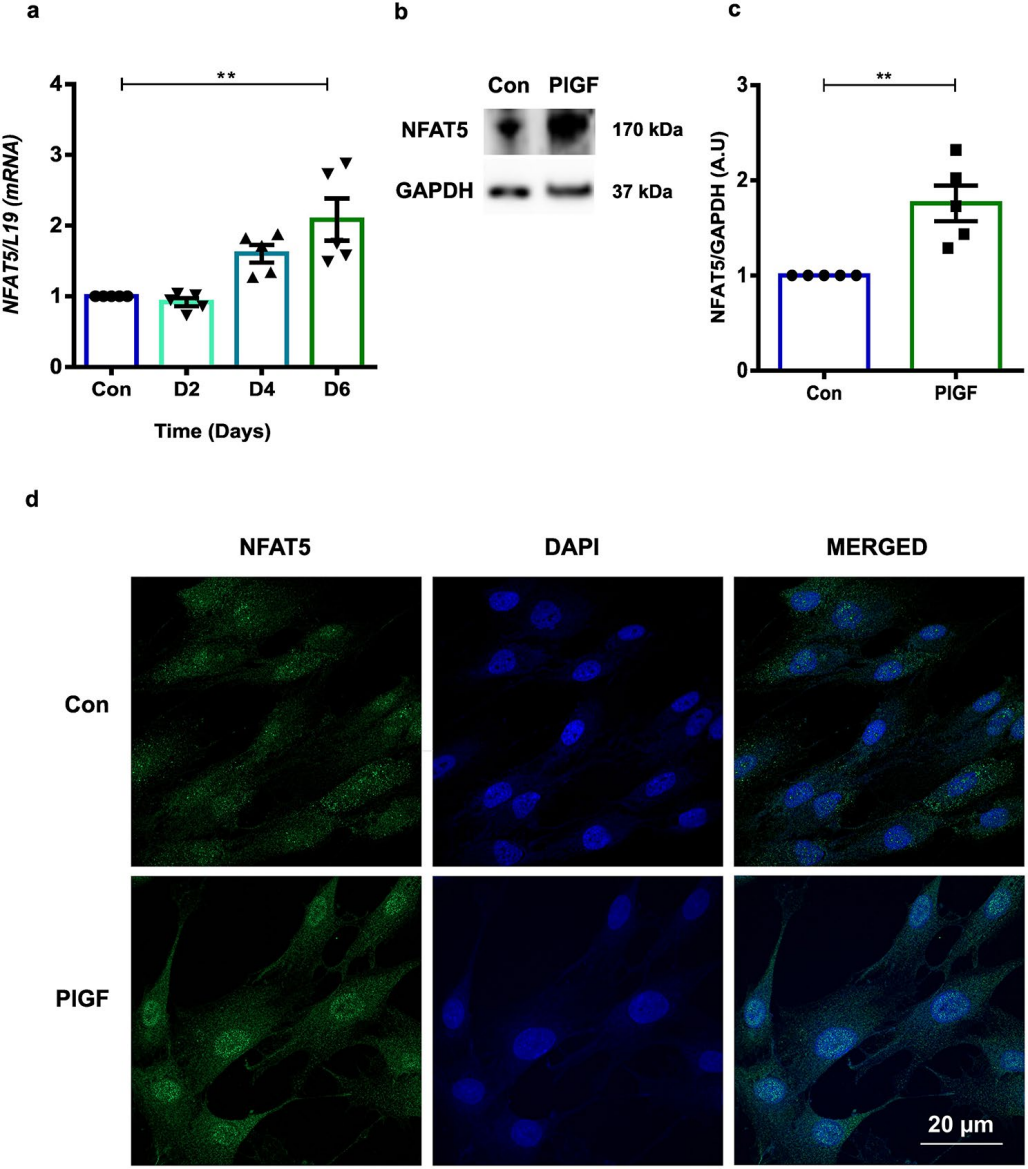
PlGF activates NFAT5 expression and activity in EnSCs. (**a**) NFAT5 mRNA transcript kinetics in EnSCs treated with PlGF for 2, 4 and 6 days at a concentration of 20 ng/ml. *L19* was used as a housekeeping gene and the data was normalized to untreated (Con) (*n* = 5, **, *p* < 0.01). (**b**) Original Western blot analysis of NFAT5 protein with GAPDH as loading control in untreated (Con) and PlGF treated EnSCs. (**c**) Average NFAT5 protein levels after 6 days treatment with PlGF (*n* = 5, ** *p* < 0.01). The samples are represented after normalization with untreated control (Con). **d**) Immunofluorescence images confirms nuclear translocation of NFAT5 from the cytoplasm when activated by PlGF (*n* = 3). Scale bar: 20 μm. Data represented as arithmetic mean ± SEM. Significance was determined using student’s unpaired two-tailed t-test with Welch’s correction method. n represents the number of independent experiments (biological replicates)

Before treatment or transfection of EnSCs, the culture medium was changed to fresh DMEM containing 2% (v/v) dextran coated charcoal stripped fetal bovine serum, 1% (v/v) antibiotic-antimycotic solution and 1% (v/v) L-glutamine for serum starvation. EnSCs were subjected to treatment with PlGF (#P1588, PlGF-1, Sigma-Aldrich) at a concentration of 20 ng/ml for 6 days [59]. The concentration was determined by a kinetic assay and time course experiments (Fig. 1 and Supplementary Fig. 1). For hyperosmolarity treatment in EnSCs, cells were treated with 800 mOsm in 2% DCC DMEM for 6 h. For gene silencing experiments, EnSCs were treated with siSGK1 (50 nM, #L-003027-00-0005, Dharmacon). The siRNAs were transfected with Lipofectamine RNAiMAX (#13,778,075, ThermoFisher Scientific) for 48 h with and without additional PlGF treatment. Stromal cell cultures were first treated with PlGF (20 ng/ml) for 4 days followed by transfection with SGK1 siRNA or in combination with PlGF for 48 h, and then continued with PlGF treatment for a further 2 days. The experimental groups are classified as Con (untreated EnSCs), PlGF, siSGK1 and siSGK1 + PlGF. Dimethyloxalylglycine, N-(Methoxyoxoacetyl)-glycine methyl ester (DMOG) (#D3695; Sigma-Aldrich) treatment in EnSCs was carried out for 24 h at a concentration of 0.5 mM.

### Conditioned medium treatment of HUVECs

Post 6 days PlGF treatment in EnSCs, cell supernatant was collected as conditioned medium (CM). The control-CM (untreated) / PlGF-CM / siSGK1 CM / siSGK1 + PlGF CM were collected respectively and stored at -80 °C until processing. HUVECs were split and plated onto cell culture plates at a density as required. Post cell adhesion, the HUVECs were treated with respective conditioned medium diluted with HUVEC growth medium at 1:1 dilution factor and incubated at 37 °C for 48 h. HUVECs were treated with Dimethyloxalylglycine, N-(Methoxyoxoacetyl)-glycine methyl ester (DMOG) (#D3695; Sigma-Aldrich) to induce hypoxia for 24 h [60]. VEGF-A (#PHC9391, ThermoFisher Scientific) treatment was carried out for 24 h at a concentration of 40 ng/ml to induce vascular permeability [61]. The experimental groups in HUVECs are classified as Con-CM, PlGF-CM, siSGK1-CM and siSGK1 + PlGF-CM.

### Quantitative real time-polymerase chain reaction (qRT-PCR)

Post treatment, cells were collected for downstream analysis of messenger RNA (mRNA) extraction and Quantitative Real-time PCR (qRT-PCR). Total RNA was extracted using TRizol™ reagent (#15,596,026, Invitrogen). One µg RNA was utilized to synthesize cDNA using the ThermoFisher Scientific Maxima™ H Minus cDNA Synthesis Master Mix with dsDNase (#M1681, Invitrogen). qRT-PCR was performed on the QuantStudio 3 Real-Time PCR System (Invitrogen) by using sets of gene-specific primers and the PowerUp™ SYBR^®^ Green Master Mix (#A25742, Invitrogen). The relative differences in PCR product amounts were quantified by the ^ΔΔ^C_T_ method, using ribosomal L19 (*L19*) as an internal housekeeping control [62]. Experiments were performed in triplicate (technical replicates). Melting curve was used to confirm amplification specificity. The gene expression levels of the samples are provided as arbitrary units defined by the ^ΔΔ^C_t_ method. All the gene-specific primers used in this study were designed using primeblast (NCBI) and purchased from Sigma-Aldrich. The primer sequence can be provided on request.

### Western blotting

Whole cell protein lysate was extracted from EnSCs cultured on 6-well plates (approx. 1 × 10^6^ at time of harvesting) using hot 1X Laemmli buffer with a cell scraper as previously reported [63]. Lamelli lysis buffer contains 0.5 M Tris hydrochloride (#9090.1, Roth) pH 6.8, 20% Sodium dodecyl sulfate (#151-21-3, Sigma-Aldrich), 0.1% Bromophenol blue (#34725-61-6, Serva),1% beta mercaptoethanol (#60-24-2, Sigma-Aldrich), and 20% glycerol (#56-81-5, Roth). Whole cell protein lysates were collected and heated at 95 °C for 3 min. Protein extracts were then loaded on to a 10% sodium dodecyl sulfate polyacrylamide gel (SDS-PAGE) using the XCell SureLock^®^ Mini-Cell apparatus (Invitrogen) followed by electrophoresis. The protein from the gel was then transferred onto a nitrocellulose membrane (#10,600,003, GE HealthCare). After incubation with 5% non-fat milk or BSA in TBST (10 mM Tris, pH 8.0, 150 mM NaCl, 0.5% Tween 20) for 60 min, the membrane was washed once with TBST and incubated with primary antibodies against NFAT5 (1:2000, #NB20-3446, Novus Biologicals) [64], SGK1 (1:1000, #07-315, Merck) [65], phospho-SGK1 (1:1000, #36 − 002, Merck) [65], p38 MAPK (1:1000, #8690S, Cell Signaling Technologies) [66], phospho-p38 MAPK (1:1000, #4511, Cell Signaling Technologies) [66], VEGF-A (1:3000, #ab46154, abcam) [67], VEGFR1 (1:1000, #2893, Cell Signaling Technologies) [68], VEGFR2 (1:1000, #2479, Cell Signaling Technologies) [69], or GAPDH (1:1000, #5174, Cell Signaling Technologies) [70] at 4 °C for overnight. Membranes were then washed three times for 15 min and incubated with HRP-conjugated anti-rabbit secondary (1:2000, #7074s, Cell Signaling Technologies) [71] antibodies for 1 h in room temperature. Post-secondary antibody incubation, blots were washed with TBST three times for 15 min and developed with the ECL system (#R-03031-D25, Advansta) according to the manufacturer’s protocols. The fluorescence signals were scanned with an iBright CL1000 (ThermoFisher Scientific), and the intensities were assessed by densitometry analysis to measure the relative expression of the target proteins using GAPDH as a loading control by ImageJ software [72].

### Immunofluorescence

For immunolabelling of cells, EnSCs (5000 cells) were seeded on 4-well glass chamber slides (#94.6170.402, Sarstedt) and cultured in 10% DCC FBS containing DMEM medium. Post treatment with PlGF as described above, the cells were fixed with 4% paraformaldehyde for 15 min, washed with PBS, and permeabilized for 15 min in 0.1% Triton X-100/PBS. The samples were then blocked with 5% BSA in 0.1% TritonX-100/PBS for 1 h at RT and washed with PBS. The slides were then incubated with primary antibodies for NFAT5 (1:200, #NB20-3446, Novus Biologicals) [64] at 4 °C overnight. Subsequently, washed with PBS and incubated with FITC conjugated secondary antibody (#4412, Alexa Fluor 488 Conjugate, ThermoFisher Scientific) for 1 h at room temperature. Post incubation, slides were washed again with PBS, dehydrated, air-dried and mounted using ProLong Gold antifade reagent containing DAPI (#P36931, Invitrogen). Fluorescence was detected with LSM 800 confocal laser scanning microscope (Zeiss). The images were captured using oil immersion, 40x objective lens. Scale bar − 20 μm. Mean fluorescence intensities were calculated using ImageJ software.

### Luciferase reporter assay

EnSCs cells were seeded onto 24-well plates at a density of 5 × 10^4^ cells/well with 10% DCC-FBS/DMEM and allowed to attach for 24 h. Post serum starvation, cells were transfected with HIF-1α vector (#87,261, Addgene) using Lipofectamine LTX with Plus reagent (#15,338,100, ThermoFisher Scientific) as per the manufacturer’s instructions. After transfection for 24 h, cells were subjected to treatment with PlGF ± siSGK1 as described above. The reporter activation was determined using the Dual-Luciferase Reporter Assay System (#E2920, Promega) according to the manufacturer’s instructions.

Briefly, growth medium was removed and cells were washed with PBS. Subsequently, cells were lysed for 15 min at room temperature using 1X passive lysis buffer. Lysed cells were used for determination of luciferase activity. LAR II reagent was added to each well, and firefly luminescence was measured using a microplate reader (LUX VARIOSKAN, ThermoFisher Scientific). Next, Stop & Glo reagent was added to each well and renilla luciferase activity was measured using a microplate reader. Three replicate wells were used for each analysis, and the results were normalized to the activity of renilla luciferase.

### ELISA

The secreted VEGF-A levels in PlGF-conditioned medium were measured with ELISA. Briefly, after the treatment of EnSCs with PlGF as described above, the conditioned medium was harvested and stored at -80 °C. The collected medium was processed with Human ELISA kit for VEGF-A (#BMS277-2, Invitrogen) following the manufacturer’s instructions performed in biologically independent experiments (with three technical replicates).

### Preparation of conditioned medium for proteomic analysis

For proteome analyses, conditioned medium (three biological replicates) was collected from EnSCs treated with and without PlGF as mentioned above. For precipitation of protein from conditioned medium, 100% acetone (ice cold): 100% MeOH (ice cold): protein solution was mixed at a ratio of 8:1:1, followed by incubation at -20 °C overnight. Post incubation the samples were washed (2X) at 2,500 g, 4 °C for 20 min. Post washing, the supernatant was then aspirated, and the pellet was air dried. After desalting using C18 stage tips, extracted peptides were separated on an Easy-nLC 1200 system coupled to a Q Exactive HFX mass spectrometer (ThermoFisher Scientific) as detailed in [73]. The peptide mixtures were separated using a 90 min segmented gradient from to 10-33-50-90% of HPLC solvent B (80% acetonitrile in 0.1% formic acid) in HPLC solvent A (0.1% formic acid) at a flow rate of 200 nl/min. The 12 most intense precursor ions were sequentially fragmented in each scan cycle using higher energy collisional dissociation (HCD) fragmentation. Acquired MS spectra were processed with MaxQuant software package version 1.6.7.0 with integrated Andromeda search engine [74]. A database search was performed against a target-decoy Homo sapiens database obtained from Uniprot, containing 103.859 protein entries and 286 commonly observed contaminants. Peptide, protein and modification site identifications were reported at a false discovery rate (FDR) of 0.01, estimated by the target/decoy approach and the fold change cut-off was set at > ± 1.0 [75] The LFQ (Label-Free Quantification) algorithm was enabled, as well as match between runs and LFQ protein intensities were used for relative protein quantification. Data analysis was performed using Perseus [76], DEP and R packages.

### BrdU cell proliferation

The effect of PlGF treated CM on HUVEC proliferation was measured using the BrdU cell proliferation assay on 96-well plates with 3000 cells (#QIA58, Sigma-Aldrich). Briefly after the treatment of HUVECs with respective CM for 48 h or/and thymidine (2 mM; #T1895, Sigma-Aldrich) for 42 h. Post treatment the cells were immunolabelled for BrdU and the cells incubated for an additional 24 h. Incorporated BrdU was detected by the BrdU Cell Proliferation Assay as instructed in the manufacture’s protocol. The experiment was performed in biologically independent experiments (with three technical replicates).

### Wound healing scratch assay

GFP-tagged HUVECs were seeded in 6-well plates at a concentration of 2 × 10^5^ cells per well. After reaching 100% confluency, HUVECs were deprived of serum for 12 h and scratched with a sterile P200 pipette tip as previously described. After removal of the debris by repeated washes, cells were subjected to respective CM treatment and scratch wound closure was closely monitored by fluorescence microscopy (EVOS M7000 cell imaging system, ThermoFisher Scientific) capturing images of the same field with a 4X objective at 0 h and 24 h. The percentage of migrated area was calculated with Image J software (v1.53k) [77]. The experiment was performed in biologically independent experiments (with three different fields of view taken for the average).

### In vitro tube formation assay

The effect of CM on angiogenic potential was assessed by the spontaneous formation of capillary-like structures by the GFP-tagged HUVECs. 96-well plates were coated with ice-cold Matrigel solution (#3533-005-02, 3533-005-02) and incubated at 37 ℃ for 60 min to allow the Matrigel to solidify. GFP-tagged HUVECs were harvested, suspended in serum-reduced (2%) endothelial growth medium, seeded in the Matrigel-coated wells at a density of 5 × 10^4^ cells/well in 100 µl of respective CM/DMOG treatment solution. Images of the tubular structures were taken using a fluorescence microscopy (EVOS M7000 cell imaging system, ThermoFisher Scientific) capturing images of the same field with a 4X objective for every 3 h. The experiment was performed in biologically independent experiments and the relative tube length and relative number of tubes formed at 24 h were calculated using an angiogenesis plugin with Image J software (v1.53k) [78].

### Endothelial barrier function study with Electrical Impedance spectroscopy (EIS)

An EIS approach was employed to study the influence of stroma secreted conditioned medium on the endothelial barrier function of HUVECs. The E16 plate (#5,469,830,001, Agilent, Germany) was mounted on to the xCELLigence RTCA (Agilent, Germany) for background reading. Later, HUVECs were trypsinized and 7000 cells per well was seeded. Respective treatment conditions with CM (technical duplicates) as mentioned above was added to the E16 plates with cells and kept for incubation at 37 °C for 30 min, for cells to equilibrate and adhere. Post cell adhesion, E16 plate containing cells was mounted on to the xCELLigence RTCA for impedance measurements. Live cell impedance was monitored every 15 min for a total period of 24 h. The experiment was performed in biologically independent experiments and the data is represented as cell index. Data acquisition and data analysis was performed using RTCA Software Pro (Agilent, Germany).

### BeWO cell invasion through endothelial monolayer with electrical impedance spectroscopy (EIS)

The invasion of BeWo through the endothelial monolayer was studied with EIS measurements. The E16 plates were coated with 0.1% gelatin and let to incubate for an hour at 37 °C. Next, 100 µl of HUVEC growth medium was added to E16 plate for background measurement. The plate was mounted on to the xCELLigence RTCA (Agilent Technologies, Germany) for background reading. Later, HUVECs were trypsinized and 5000 cells per well were added to the E16 plates and kept for incubation at 37 °C for cells to equilibrate and adhere. Post 24 h, the cells were treated with respective treatment conditions with CM (Con / PlGF / siSGK1 / siSGK1 + PlGF) as mentioned above for 48 h. Ten hours prior treatment end point, E16 plates containing cells were clamped again onto the xCELLigence RTCA and placed in the incubator at 37 °C. Live cell impedance was monitored every 15 min for a total period of 10 h to ensure stromal cell monolayer formation.

Post treatment time point, the cell index measurement was paused and 2500 BeWo cells per 100 µl of 10% FBS DMEM were added. EIS cell index measurement was continued to monitor to the BeWo invasion through CM treated HUVEC monolayer. Live cell impedance was monitored every 30 min for a total period of 8 h. The experiment was performed in biologically independent experiments and the data is represented as normalized cell index relative to the time point BeWo cells were added to the HUVEC monolayer. Data acquisition and data analysis was performed using RTCA Software Pro (Agilent Technologies, Germany).

### In silico data analysis

*In silico* analysis was performed on an open-access gene-expression data platform. The gene expression of *NFAT5* was verified by analysis of the normal endometrium at distinct phases of the menstrual cycle (*GDS2052*) [41]. To investigate the significance of *NFAT5* in PE pathogenesis, we obtained its gene expression data in human decidua of pre-symptomatic preeclamptic women and healthy pregnant women (*GDS3467*) [43].

### Statistical analysis

Data are presented here as means ± SEM. n represents the number of independent experiments (biological replicates). All treatment groups are normalized with their respective control groups in EnSCs (Con) and HUVECs (Con-CM) respectively. Data represented were analysed for significance using student’s unpaired two-tailed t-test with Welch’s correction approach and One-way ANOVA. Results with *p* < 0.05 were considered statistically significant. Figures and statistical analysis were made in Graphpad Prism (v 7.0) and Inkscape (v 1.3).

## Results

### PlGF drives tonicity independent activation of NFAT5 in endometrial stromal cells

We first explored the spatio-temporal expression of *NFAT5* in the endometrium. Expression of *NFAT5* (Supplementary Fig. 1a) was found to be higher in the proliferative phase compared with the late secretory phase of the menstrual cycle (*GEO 2052*) [41]. Single cell analysis confirmed high *NFAT5* expression in the stromal population among the other endometrial cell types and extra villous trophoblast (Supplementary Fig. 1b & c). According to the Human Protein Atlas, NFAT5 is expressed throughout the endometrium and staining was highest in the perivascular area and blood vessels [79] (Supplementary Fig. 1d). We next assessed the involvement of NFAT5 in the etiology of PE pathogenesis. To address this, we manually mined expression levels of *NFAT5* obtained from gene expression studies of the first trimester decidua prior to the onset of PE compared with healthy pregnancies (*GEO 3467*) [43]. We found that *NFAT5* transcripts were upregulated in the decidua of pre-symptomatic women who developed PE later (Supplementary Fig. 1e). Taken together, the *in-silico* analysis reveals that endometrial NFAT5 expression is highest in the proliferative phase and is associated prior to the onset of PE.

PlGF is a member of the VEGF superfamily and aberrant expression is associated with abnormal blood vessel physiology *via* NFAT5 [52]. Thus, we sought to determine a putative link between PlGF on NFAT5 regulation in endometrial cells. EnSCs were treated with varying concentrations (titration of 2.5–50 ng/ml) of PlGF for 6 days. As illustrated in Supplementary Fig. 2a, *NFAT5* mRNA expression was highly upregulated by PlGF at a concentration of 20 ng/ml compared with the other concentrations tested. The time kinetics study revealed gene expression of *NFAT5* when EnSCs were treated with PlGF (20 ng/ml) were highest after 6 days of treatment (Fig. 1a). All proceeding experiments used PlGF at a concentration of 20 ng/ml for a treatment period of 6 days in EnSCs. Consistent with our mRNA data, PlGF also significantly upregulated NFAT5 protein levels in parallel (Fig. 1b-c and Supplementary Fig. 2b). Further, we examined PlGF activated NFAT5 spatial regulation in EnSCs using an immunofluorescence approach. Endogenous NFAT5 was localized to the cytosol in untreated EnSCs and was found to be translocated to the nucleus upon PlGF treatment (Fig. 1d). NFAT5 is well recognized to be activated under hyperosmolarity cellular stress (Hyp Osm), we compared its transcriptional activity in EnSCs by treating with Hyp Osm (800 mOsm/ml) medium for 3 h as a positive control (Supplementary Fig. 3a-c). Thus, we observed that PlGF can activate NFAT5 in EnSCs in a *tonicity independent* manner.

### p38 MAPK regulated NFAT5 activation upregulates SGK1 expression in endometrial stromal cells

**Fig. 2:**
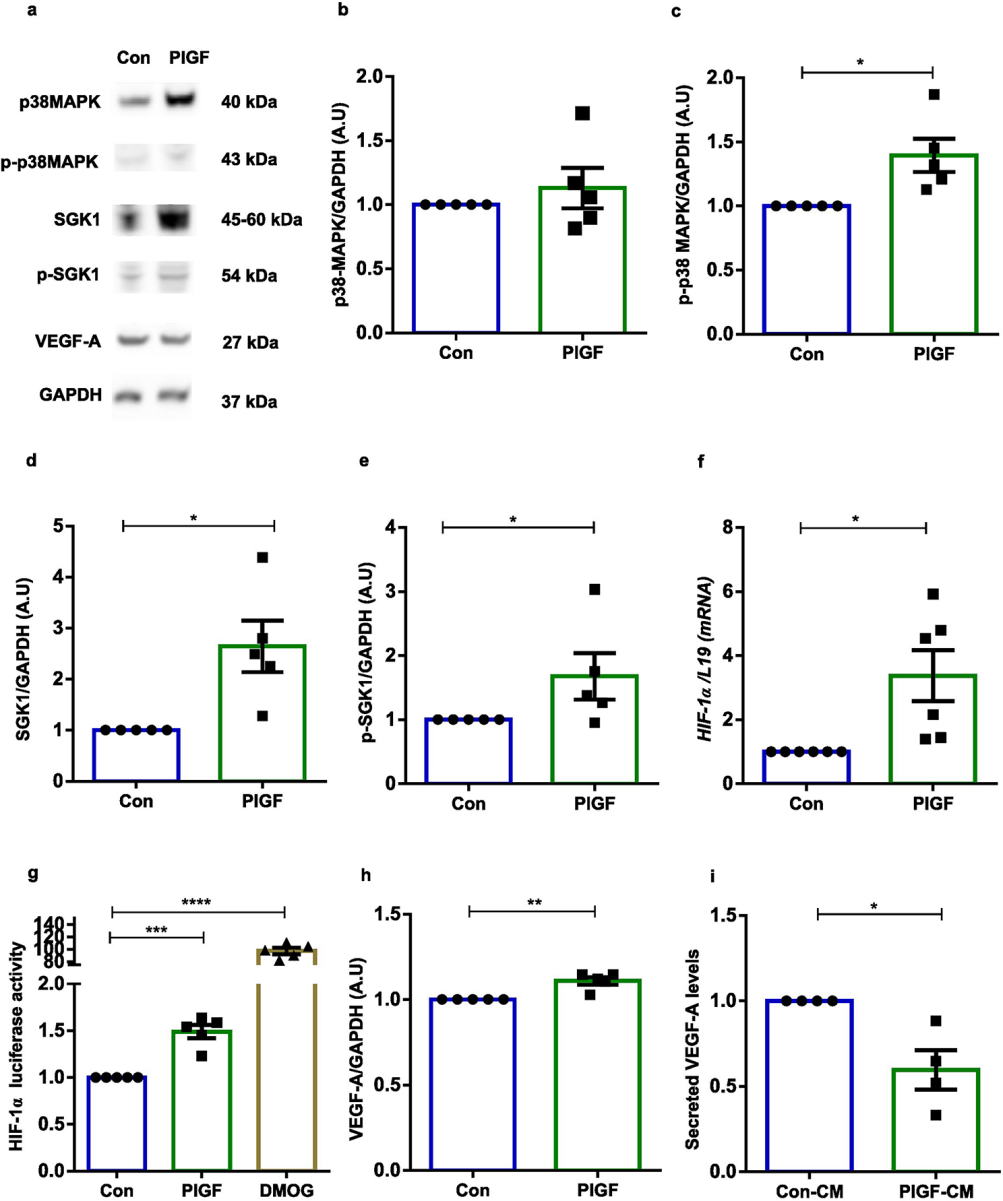
PlGF-NFAT5 angiogenic signaling axis in EnSCs. (**a**) Original Western blots of total and phosphorylated levels of p38 MAPK, SGK1 and total VEGF-A targets with GAPDH as loading control in untreated (Con)and PlGF treated EnSCs. (**b**-**e**) Average protein expression levels of total and phosphorylated levels of p38 MAPK and SGK1 targets in untreated (Con) and PlGF treated EnSCs (*n* = 5, *, *p* < 0.05). (**f**) qPCR analysis of *HIF-1α* transcript levels in untreated (Con) and PlGF treated EnSCs. *L19* was used as a housekeeping gene (*n* = 5, *, *p* < 0.05). (**g**) Luciferase reporter assay measuring the HIF-1α promoter activity in untreated (Con), PlGF and DMOG (positive control for hypoxia, 0.5 mM for 24 h) treated EnSCs (*n* = 5, ***, *p* < 0.001, ****, *p* < 0.0001). (**h**) Immunoblotting showing average protein expression levels of VEGF-A (Con) in untreated and PlGF treated EnSCs (*n* = 5, **, *p* < 0.01). (**i**) Supernatant from untreated (Con) and PlGF treated EnSCs was collected and secreted VEGF-A levels were quantified with ELISA (*n* = 5, *, *p* < 0.05). Data represented here as arithmetic mean ± SEM. The treatment samples groups (PlGF) are represented after normalization with untreated control (Con). Significance was determined using student’s unpaired two-tailed t-test with Welch’s correction method. n represents the number of independent experiments (biological replicates)

p38 Mitogen-Activated Protein Kinase (p38 MAPK), is known to activate NFAT5 transcriptional activation under hypertonic stimulus [80]. To verify the participation as an upstream target of NFAT5, we examined protein levels of total and phosphorylated levels of p38 MAPK following treatment with PlGF. Figure 2a-c and Supplementary Fig. 4 demonstrates the increased levels of total p38 MAPK protein, with significant upregulation of phosphorylated levels of p38MAPK. NFAT5 is a regulator for various angiogenic mediators and factors including; SGK1, HIF-1α and VEGF-A [52]. Next a further series of experiments tested if PlGF indeed contributed to the activation of these downstream targets. Our findings show both total and phosphorylated SGK1 protein levels were significantly upregulated in PlGF treated stromal cells (Fig. 2a, d-e). SGK1 is a known stimulator of HIF-1α, a known modulator in angiogenic signaling [81, 82]. Elevated levels of *HIF-1α* transcripts were observed with PlGF treatment in stromal cells (Fig. 2f). Additionally, PlGF exerted a strong stimulating effect on HIF-1α promoter activity (luciferase) in EnSCs (Fig. 2g). DMOG treatment was used as positive control for HIF-1α promoter activation (0.5 mM for 24 h) [60].

To further validate the angiogenic pathway mediated by NFAT5 activation, we verified the intracellular and secreted protein levels of pro-angiogenic factor, VEGF-A. PlGF mediated NFAT5 stimulation significantly increased cellular VEGF-A protein levels in EnSCs (Fig. 2a-h). Surprisingly, the secreted protein levels of VEGF-A (Fig. 2i) in EnSCs supernatant (CM) was decreased compared with untreated control levels. These data identify the aberrant PlGF mediated angiogenic signaling in EnSCs with a decrease in secreted proangiogenic factor VEGF-A.

### Secretome analysis reveals that PlGF treated CM is associated with pathological angiogenic signaling

**Fig. 3:**
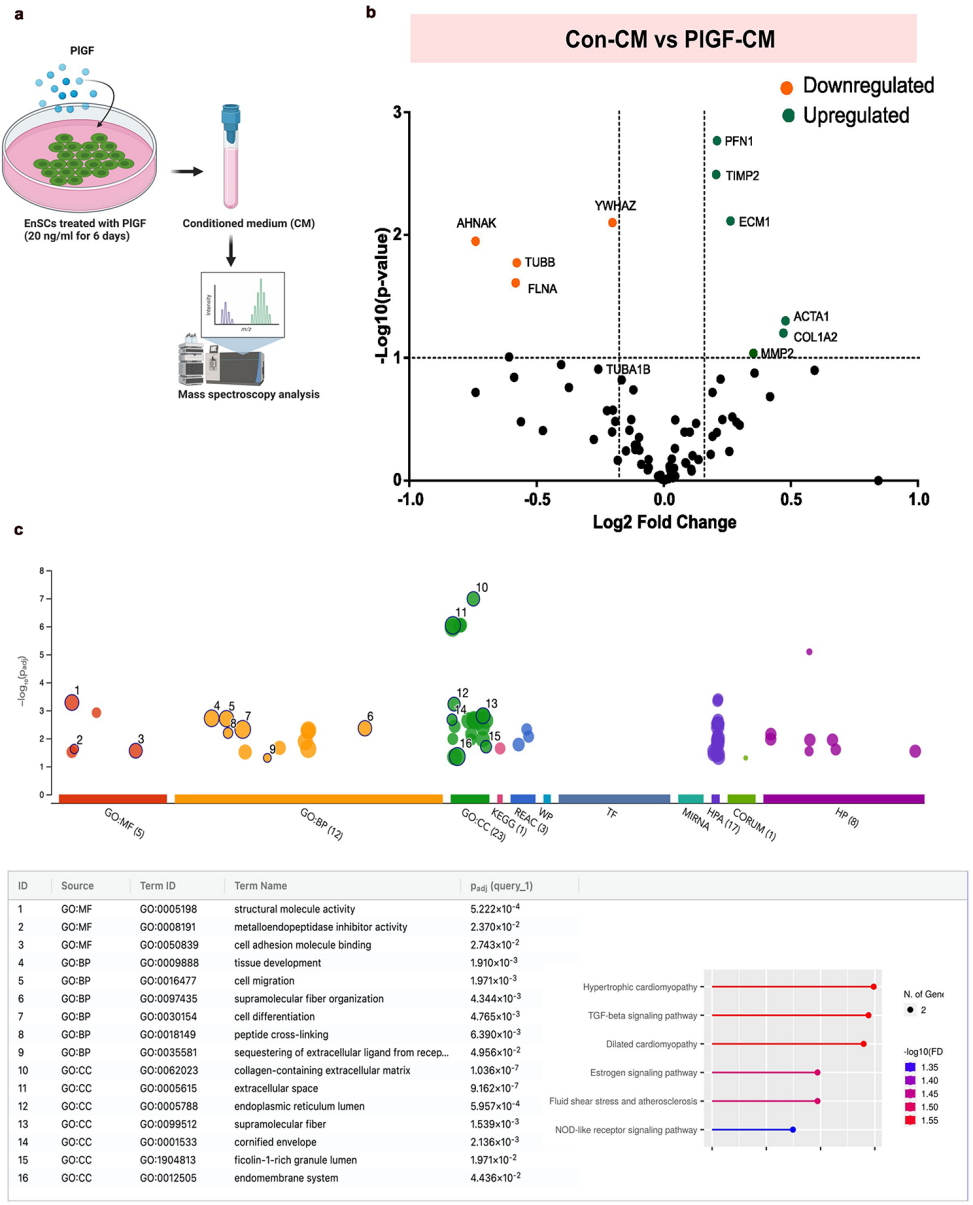
Proteomics profile of PlGF treated conditioned medium (CM) from EnSCs. (**a**) Schematics describing the experimental approach of mass spectrometry analysis on CM (supernatant) collected from EnSCs post 6 days treatment without (Con) and with PlGF (20 ng/ml). (**b**) Volcano plot showing significantly upregulated (green) and downregulated (orange) proteins in PlGF treated CM from EnSCs. Each point represents one protein; black points are the rest of the proteins obtained in the global proteomic analysis. The significance threshold range is 0.05 and the fold change threshold is -1 and + 1. (**c**) GO enrichment analysis of the protein signature depicting the enriched biological process and pathways associated with PlGF treated CM in EnSCs. n represents the number of independent experiments (biological replicates)

To investigate if PlGF can dysregulate other angiogenic mediators in EnSCs, we sought to understand its comprehensive and global effect on stromal cell secreted factors. We utilized proteomics to characterize the supernatant (CM) from EnSCs. Liquid chromatography mass spectrometry (LC-MS) analysis was performed by comparing the protein cargo present in Con-CM (*n* = 3) and PlGF-CM (*n* = 3) collected from EnSCs post 6 days treatment with and without PlGF as described in Fig. 3a. This comparison revealed a total of 68 dysregulated secreted proteins. Differentially regulated proteins were shown in volcano plots as seen in Fig. 3b. A total of 36 upregulated and 32 downregulated differentially regulated proteins were identified in the CM as being associated with PlGF treatment in the EnSCs. Some of the significantly upregulated proteins (green) in PlGF-CM were actin and extra cellular matrix (ECM) associated components such as ECM-1, ACTA1, PFN1, COL1A2, MMP2 and inhibitors of the matrix metalloproteinases such as TIMP2. The significantly downregulated proteins (orange) include AHNAK, FLNA and YWHAZ. Other ECM associated proteins such as COL2A6, COL6A3, and COL3A1 were not differentially expressed based on the differential expression thresholds we used in this study, however we observed modest but significant changes in their expressions too. Gene Ontology (GO) analysis of the protein signature associated with supernatant from the PlGF treated cells identified pathways associated with structural remodelling, ECM modification and pathological vessel development (Fig. 3c). Thus, the proteomic analysis points to an anti-angiogenic signature mediated by PlGF.

### HUVECs display abnormal ‘hypersprouting’ behaviour when treated with conditioned medium (CM)

**Fig. 4:**
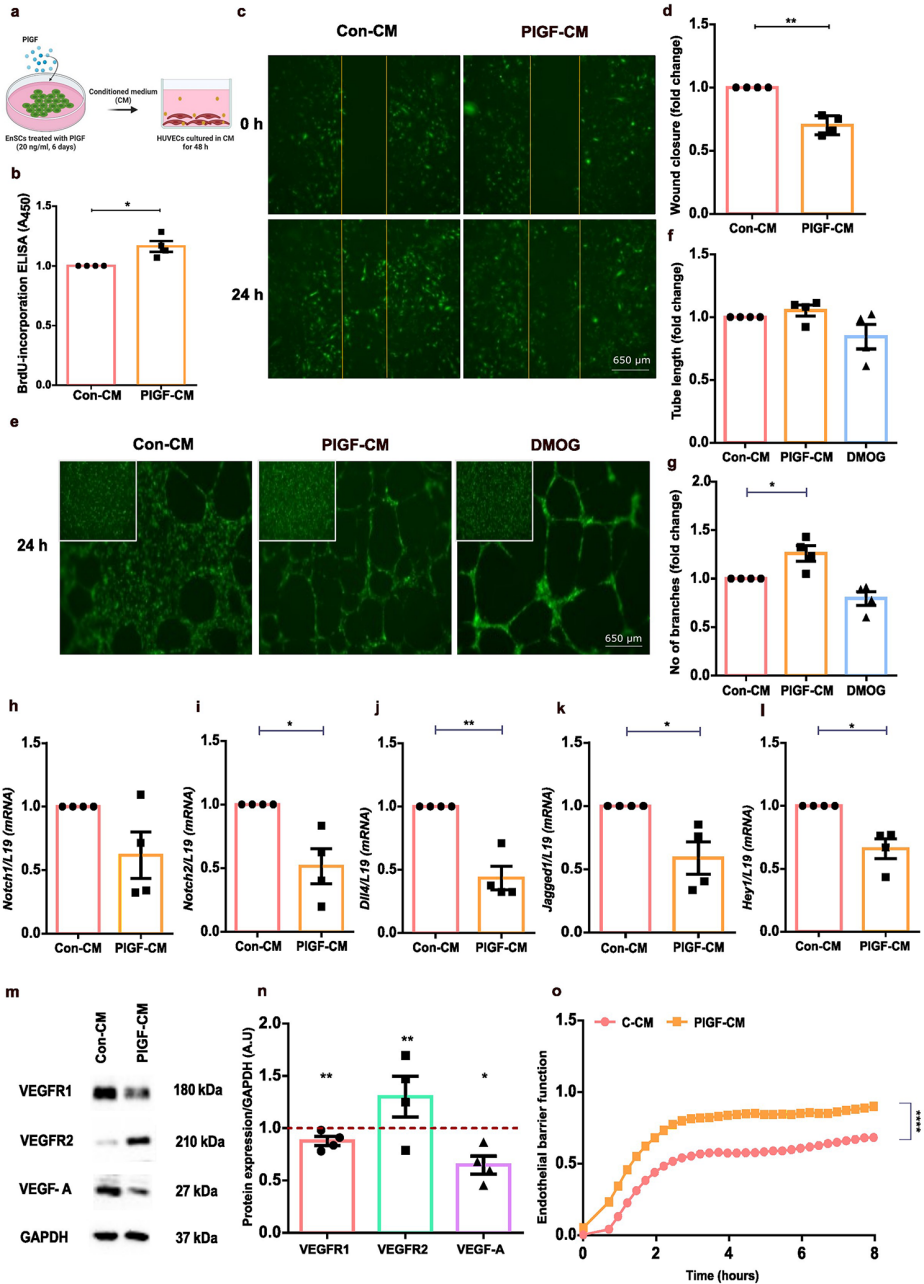
Angiogenic effect of PlGF-NFAT5 signaling axis on HUVECs. (**a**) Schematics describing the experimental approach of CM treatment on HUVECs. (**b**) BrdU incorporated ELISA analysis for cell proliferation measured in Con-CM and PlGF-CM treated HUVECs (*n* = 4, *, *p* < 0.05). (**c**) Representative fluorescence microscopic images of wound healing scratch assay on Con-CM and PlGF-CM treated HUVECs at 0 and 24 h (*n* = 4). Yellow line represents the wound area created. Scale bar: 650 μm. (**d**) Wound closure rate in Con-CM and PlGF-CM treated HUVECs at 24 h (*n* = 4, **, *p* < 0.01) explain normalization. (**e**) Representative fluorescence microscopic images of tube formation assay on a matrigel with Con-CM, PlGF-CM and DMOG (positive control; 0.5 mM for 24 h) treated HUVECs at 24 h (*n* = 4). The insert displays HUVECs seeded on the matrigel at 0 h. Scale bar: 650 μm. (**f**) Tube formation assay analysis showing tube length in Con-CM, PlGF-CM and DMOG treated HUVECs at 24 h (*n* = 4). (**g**) Tube formation assay analysis depicting number of branches in Con-CM, PlGF-CM and DMOG treated HUVECs at 24 h (*n* = 4, *, *p* < 0.05). (**h**-**l**) qPCR analysis of Notch receptors (*Notch 1 and Notch 2*), ligands (*Dll4 and Jagged-1*) and target genes (*Hey 1*) in Con-CM and PlGF-CM treated HUVECs. *L19* was used as a housekeeping control. (*n* = 4, *, *p* < 0.05, **, *p* < 0.01). (**m**) Original Western blots of VEGFR1, VEGFR2 and VEGF-A targets with GAPDH as loading control in Con-CM and PlGF-CM treated HUVECs. (**n**) Average protein levels of VEGFR1, VEGFR2 and VEGF-A in Con-CM and PlGF-CM treated HUVECs (*n* = 4, *, *p* < 0.05, **, *p* < 0.01). Data represented here as arithmetic mean ± SEM. The treatment samples groups (PlGF-CM) are represented after normalization with control (Con-CM). Significance was determined using student’s unpaired two-tailed t-test with Welch’s correction method. (**o**) EIS analysis of cell impedance values in Con-CM and PlGF-CM treated HUVEC monolayer representing endothelial barrier function (*n* = 4, ****, *p* < 0.0001). Significance was determined using student’s unpaired two-tailed t-test with Welch’s correction method for cell impedance values at 4 h. n represents the number of independent experiments (biological replicates)

Pathological PlGF is associated with poor vessel development in the retina [83]. We postulated that the paracrine signaling from PlGF treated endometrial cells would also support poor vessel formation. To test this conjecture, HUVECs were treated with CM collected from untreated or PlGF treated EnSCs (Fig. 4a). We then verified its effects on angiogenic checkpoints in HUVECs employing different functional assays. As seen in Fig. 4b, PlGF-CM significantly increased cell proliferation in HUVECs as evidenced with the BrdU ELISA assay. Further, a wound healing scratch assay with GFP-HUVECs showed reduced cell migration upon PlGF-CM treatment (Fig. 4c-d) and an in vitro tube formation assay showed no significant change in tube length between Con-CM and PlGF-CM treatment on HUVECs (Fig. 4e-f). However, PlGF-CM treated HUVECs displayed a greater number of endothelial cell branches pointing to an abnormal ‘hypersprouting’ behaviour (Fig. 4e-g). Hypersprouting behaviour in endothelial cells is a phenotype of deregulated Notch signaling [84]. To investigate Notch signaling in PlGF-CM mediated anti-angiogenic behaviour seen in HUVECs, gene expression levels of Notch signaling effectors such as Notch receptors (*Notch 1 and Notch 2*), ligands (*Dll4 and Jagged-1*) and target genes (*Hey 1 and Hes1*) were downregulated with PlGF-CM treated HUVECs (Fig. 4h-l and Supplementary Fig. 5a). Further, to substantiate the increase of hypersprouting and notch downregulation, we verified the protein levels of VEGF receptors and VEGF-A levels in HUVEC after CM treatment. PlGF-CM treated HUVECs showed enhanced VEGFR2 protein expression with downregulated levels of VEGFR1 and VEGF-A (Fig. 4m-n and Supplementary Fig. 5b). Thus, these results strongly suggest that PlGF-CM modulate hypersprouting in HUVECs *via* repression of notch signaling and upregulation of VEGFR2. Regulation and maintenance of the endothelial barrier during angiogenesis is critical for end organ function [85]. The influence of the stromal secreted factors on endothelial barrier function regulating vascular resistance and permeability is studied with an EIS approach. EIS measurements revealed an increase in cell index in HUVECs with PlGF-CM treatment, demonstrating an increase of junctional resistance and decrease in permeability between endothelial cells (Fig. 4o). Together, these data indicate that dysregulated PlGF-NFAT5-SGK1 signaling in stroma mediate negative angiogenic effects on HUVECs with an altered secretome signature.

### Silencing of SGK1 improves secreted VEGF-A levels in EnSCs

**Fig. 5:**
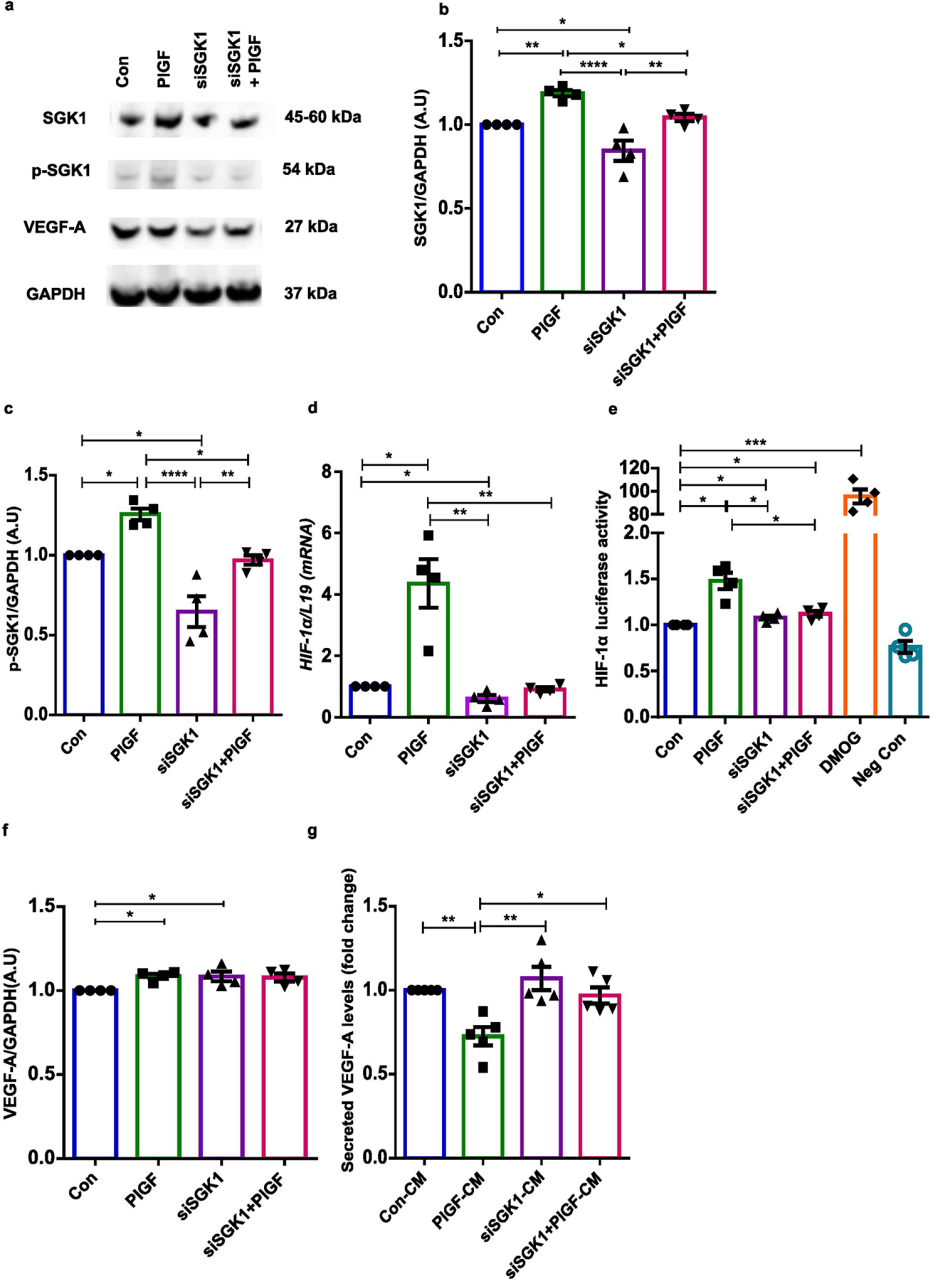
Inhibition of SGK1 gene expression in EnSCs. (**a**) Original Western blots of total and phosphorylated levels of SGK1 and total VEGF-A targets with GAPDH as loading control in untreated (Con), PlGF and siSGK1 ± PlGF EnSCs. (**b**-**c**) Average protein levels of total and phosphorylated SGK1 in untreated (Con), PlGF and siSGK1 ± PlGF treated EnSCs (*n* = 4, * *p* < 0.05, ** *p* < 0.01, ***, *p* < 0.001, ****, *p* < 0.0001). (**d**) qPCR analysis of *HIF-1α* transcript levels in untreated (Con), PlGF and siSGK1 ± PlGF treated EnSCs (*n* = 4, * *p* < 0.05, ** *p* < 0.01). *L19* was used as a housekeeping control. (**e**) Luciferase reporter assay analysis of HIF-1α promoter activity in untreated (Con), PlGF, siSGK1 ± PlGF and DMOG (57 nM) treated EnSCs (*n* = 4, * *p* < 0.05, ***, *p* < 0.001). (**f**) Average protein levels of VEGF-A in untreated (Con), PlGF and siSGK1 ± PlGF treated EnSCs (*n* = 4, * *p* < 0.05, ** *p* < 0.01). (**g**) ELISA analysis measuring secreted VEGF-A protein levels in CM from untreated (Con), PlGF and siSGK1 ± PlGF treated EnSCs (*n* = 4, * *p* < 0.05, ** *p* < 0.01). Data represented here as arithmetic mean ± SEM. The treatment samples groups (PlGF/siSGK1/siSGK + PlGF) are represented after normalization with control (Con). Significance was determined using student’s unpaired two-tailed t-test with Welch’s correction method and One-way ANOVA. n represents the number of independent experiments (biological replicates)

Endometrial SGK1 plays a paramount role in endometrial physiology and for the maintenance of pregnancy. We therefore examined whether SGK1 is the key ‘check point’ molecule driving the stroma-endothelial paracrine pathway upon PlGF stimulation. SGK1 gene silencing was efficient, suppressing both total and phosphorylated SGK1 protein expression levels in EnSCs (Fig. 5a-c and Supplementary Fig. 6).

Similarly, SGK1 inhibition with PlGF again paralleled the effect on gene silencing, significantly downregulating both total and phosphorylated levels of SGK1 protein (Fig. 5a-c). Strikingly, silencing of SGK1 transcripts was also followed by significant decrease in *HIF-1α* mRNA levels and promoter activity in EnSCs (Fig. 5d-e). The effect was mimicked in EnSCs treated with siSGK1 + PlGF as well (Fig. 5d-e). DMOG treatment in EnSCs was used as a positive control (0.5 mM for 24 h). Intriguingly, attenuated SGK1 expression in EnSCs did not significantly alter total VEGF-A levels when using western blotting (Fig. 5a-f). However, SGK1 silencing improved secreted VEGF-A levels in the CM (Fig. 5g) and the VEGF-A protein signature (total and secreted) remained the same also in sample groups where siSGK1 inhibition was combined with PlGF treatment in EnSCs (Fig. 5a, f-g). These results confirm the key molecular role of SGK1 in the angiogenic communication pathway.

#### Inhibition of endometrial stromal SGK1 improves angiogenic behaviour of HUVECs

**Fig. 6:**
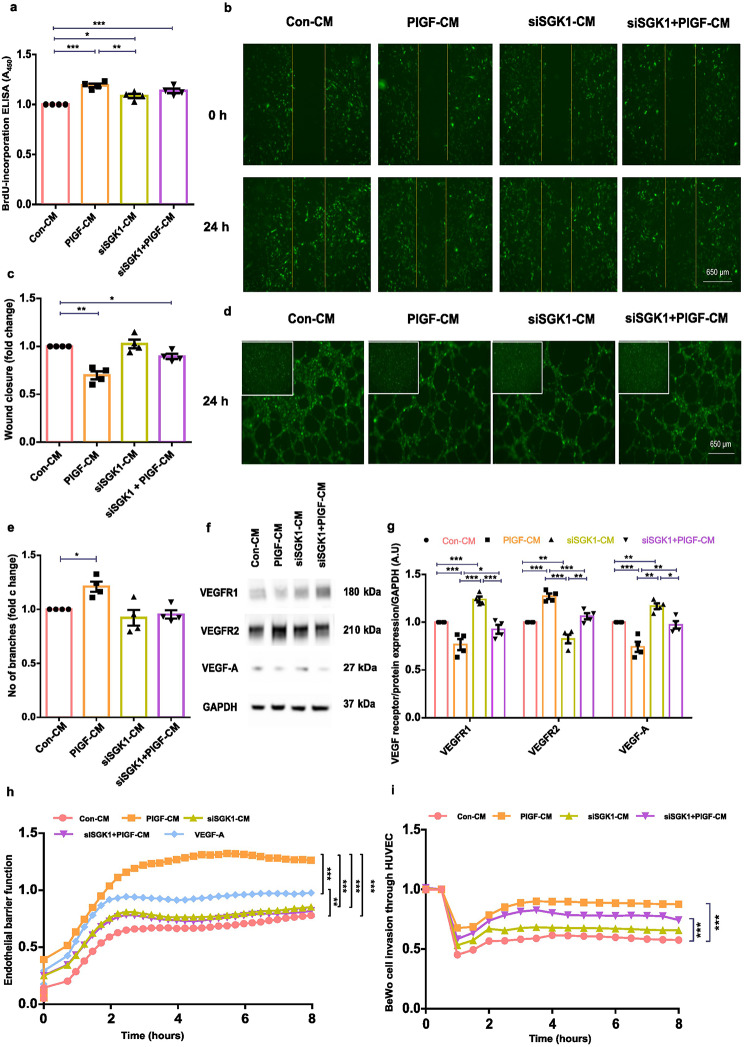
Silencing of SGK1 improved angiogenic effects in HUVECs. (**a**) BrdU ELISA analysis of cell proliferation in Con-CM, PlGF-CM and siSGK1 ± PlGF-CM treated HUVECs (*n* = 4, *, *p* < 0.05, **, *p* < 0.01). (**b**) Representative fluorescence microscopic images of wound healing scratch assay on Con-CM, PlGF-CM and siSGK1 ± PlGF-CM treated GFP-HUVECs at 0 and 24 h (*n* = 4). Yellow line represents the wound area created. Scale bar: 650 μm. (**c**) Wound scratch assay analysis showing wound closure rate in Con-CM, PlGF-CM and siSGK1 ± PlGF-CM treated GFP-HUVECs analysed with ImageJ at 24 h (*n* = 4, *, *p* < 0.05, **, *p* < 0.01). (**d**) Representative fluorescence microscopic images of tube formation assay on a matrigel with Con-CM, PlGF-CM and siSGK1 ± PlGF-CM treated GFP-HUVECs at 24 h (*n* = 4). The insert displays GFP-HUVECs seeded on the matrigel with respective treatment condition at 0 h. Scale bar: 650 μm. (**e**) Tube formation analysis showing number of branches in Con-CM, PlGF-CM and siSGK1 ± PlGF-CM treated GFP-HUVECs at 24 h (*n* = 4, *, *p* < 0.05). (**f**) Original western blots of VEGFR1, VEGFR2 and VEGF-A targets with GAPDH as loading control in Con-CM, PlGF-CM and siSGK1 ± PlGF-CM treated HUVECs. (**g**) Average protein expression levels of VEGFR1, VEGFR2 and VEGF-A in Con-CM, PlGF-CM and siSGK1 ± PlGF-CM treated HUVECs (*n* = 4, *, *p* < 0.05, **, *p* < 0.01, ***, *p* < 0.001). Data represented here as arithmetic mean ± SEM. The treatment samples groups (PlGF-CM/siSGK1-CM/siSGK + PlGF-CM) are represented after normalization with control (Con-CM). Significance was determined using student’s unpaired two-tailed t-test with Welch’s correction method and One-way ANOVA. (**h**) EIS analysis of cellular impedance in Con-CM, PlGF-CM, siSGK1 ± PlGF-CM and VEGF-A (40 ng/ml) treated HUVEC monolayer representing endothelial barrier function. The measurement reads for 8 h post CM treatment are represented here (*n* = 4, ***, *p* < 0.001). (**i**) EIS analysis of BeWo cell migration through HUVEC monolayer. Data represented here as normalized cell impedance values with respective to the BeWo addition time point to the HUVEC monolayer. The measurement reads for 8 h post BeWo addition are represented here (*n* = 4, ***, *p* < 0.001, ****, *p* < 0.0001). Significance was determined using student’s unpaired two-tailed t-test with Welch’s correction method for cell impedance values at 4 h. n represents the number of independent experiments (biological replicates)

To explore the functional relevance of SGK1 in mediating the paracrine mechanism, HUVECs were treated with CM from EnSCs with SGK1 inhibition with and without PlGF treatment. siSGK1 ± PlGF-CM decreased cell proliferation and improved cell migration behaviour in HUVECs as seen with Con-CM (Fig. 6a -c). Further, the tube formation ability in HUVECs was improved both with siSGK1 ± PlGF-CM with decrease in number of branches observed (Fig. 6d-e). siSGK1 ± PlGF-CM treatment in HUVECs also showed an increase in notch receptors (*Notch 1 and Notch 2*), ligands (*Dll4 and Jagged-1*), and target genes (*Hey 1 and Hes1*), confirming rescue of notch signaling function upon SGK1 inhibition (Supplementary Fig. 7). With upregulation of notch signaling, the protein expression levels of VEGFR1/2 and VEGF-A in HUVECs were reversed with siSGK1 ± PlGF-CM (Fig. 6f-g and Supplementary Fig. 8). Thus, these data confirm SGK1 inhibition in EnSCs improves secreted angiogenic cues, attenuating the hypersprouting phenotype in HUVECs. In addition, cellular impedance analysis with EIS showed improved endothelial barrier function with increase in cell permeability under the influence of both siSGK1 ± PlGF-CM (Fig. 6h). HUVECs were treated with VEGF-A (40 ng/ml for 24 h) as positive stimulator for permeability.

Trophoblast invasion of the maternal blood vessels is critical for development of the placenta. Shallow or poor invasion of the maternal blood vessels by is associated with PE [86]. We postulated that aberrant PlGF signaling in the endometrial stromal in turn produces cues to prevent adequate trophoblast invasion. As seen in Fig. 6i, the BeWo invasion was impeded and associated with high resistance (i.e. higher cell index) in PlGF-CM treated HUVECs compared with Con-CM. Interestingly, SGK1 inhibition improved the PlGF driven poor BeWo invasion through the HUVEC monolayer as measured by the decrease in cell impedance values (Fig. 6i). Together, these findings emphasis the important role of stromal SGK1 regulation in mediating angiogenic stimulus during uterine angiogenesis.

## Discussion

The physiological changes in the endometrium during the menstrual cycle are associated with profound angiogenesis [87]. Any impairment in endometrial milieu or disruptions in maternal vessel adaptations is considered to increase the risk of adverse pregnancy outcomes [5–7]. PE is characterized by abnormal spiral artery remodelling, angiogenic imbalance, defective placentation, placental ischemia, and oxidative stress at the maternal-fetal interface, thereby resulting in maternal endothelial dysfunction and end-organ damage [14, 15, 88]. Regardless of numerous proposed mechanisms, the underlying cause for PE is still unclear. Recently, several studies ratified the influence of maternal decidua in PE progression [14–16]. Gomez et al., identified transcriptomic alterations associated with defective decidualization in the endometrium from patients with a history of severe PE [16]. Similarly, Sufriyana et al., reported that endometrial maturation was abnormal prior to the onset of PE [89].

In our recent study, we also report a pathological role of endometrial PlGF contributing to an altered pre-pregnancy maternal microenvironment, by upregulating Rac1-PAK1 signaling axis resulting in increase of cell stiffness [40]. These studies emphasize the pre-pregnancy decidual contribution in PE pathogenesis and highlights the need for more prospective studies on endometrial health. In line with this, we hypothesized that the pathophysiological manifestation for poor placentation likely occurs well before pregnancy, possibly involving an abnormal endometrial vasculature. A variety of hormones, growth factors and cytokines participate in normal vascular development post menstruation and in vessel adaptation during decidualization [19, 23, 24, 90]. Many of these factors when present at abnormal levels could lead to endothelial dysfunction by inhibiting key signaling events and chronically promoting poor uterine vessels at the maternal site prior to pregnancy [19, 25]. Particularly, in this study we aimed to decipher the pathological role of endometrial PlGF in uterine vessel development during endometrial regeneration. PlGF, a known VEGF homolog is selectively associated with pathological angiogenesis and inflammation [27, 29, 30]. PlGF is well characterized for its role on blood vessels, but its effects on non-vascular cells is not well known [30, 33]. PlGF is temporally and spatially regulated in the endometrium, localized to glandular and luminal epithelial cells, with staining in the stromal cells surrounding the maternal spiral arteries [26]. Hence, aberrant local production of PlGF in the surrounding stroma may have functional implications in endometrial vascularization and in spiral artery remodelling during early pregnancy events.

NFAT5 was identified as a regulator of cellular osmoadaptive responses under hypertonic stress conditions [91]. Besides its osmosensitive function, various reports demonstrate its potential protective role under non-hypertonic activation [46]. NFAT5 transcriptional functioning associated with deregulated pro-angiogenic factors is described in different pathogenesis. Yu et al., reported a positive correlation of NFAT5 expression in the highly vascularized glioblastoma tumours [45]. Increased NFAT5 activity enhanced glioblastoma cell-driven angiogenesis by mediating secretion of EGF like domain multiple 7 (EGFL7) via the miR-S38-3p axis [45]. High-salt mediated NFAT5/STAT3 signaling activation in breast cancer cell aids in proliferation and migration through activation of angiogenic factor VEGF-A [92]. In a similar study, Hollborn et al., suggest that hyperosmolarity stress induced NFAT5 stimulation aggravates neovascularization and oedema formation in retinal pigment epithelial cells via PlGF/VEGF-A signaling effectors [52]. In contrast, the pathological role of isotonic NFAT5 activation is poorly understood. Specifically, the expression kinetics of NFAT5 in endometrium and its potential role in uterine angiogenesis remain unexplored. The current study unravels the distinct regulation of tonicity independent NFAT5 activation in the endometrium. In EnSCs, PlGF induced NFAT5 mRNA and increased its nuclear abundance confirming its transcriptional activation. Additionally, the increase of NFAT5 immunoreactivity in endometrial tissues during the proliferative phase of the cycle in the perivascular region around the blood vessels emphasizes its importance in influencing uterine vessel formation.

p38 MAPK is one of the few kinases that is involved in regulating both nuclear translocation and in mediating transcriptional activation of NFAT5 [93, 94]. We report here that PlGF induces NFAT5 transcriptional activation in EnSCs through p38 MAPK signaling. NFAT5 has been reported to be a DNA binding transcriptional activator that controls various angiogenic genes such as SGK1, COX2 and VEGF-A [52, 95]. We show that increased NFAT5 transcription in EnSCs leads to enhanced SGK1 protein phosphorylation, thereby further positively mediating the angiogenic downstream signaling. In keeping with this finding Wang et al. have shown that increased placental SGK1 is associated with PE [96].

SGK1 downstream targets include HIF-1α and NF*k*B, which are known mediators in angiogenic signaling [54, 57, 58]. Since hypoxia is a well-known stimulus that promotes vessel growth [97, 98], we examined the role of this crucial molecular downstream target of SGK1. Our findings show enhanced levels of HIF-1α transcripts and increased promoter activity in EnSCs upon PlGF stimulation. Hypoxia is known to regulate various pro-survival pathways affecting VEGF secretion in vascular pathophysiology [98–100]. Hence, we further unravelled the signaling axis reporting increased cellular levels of pro-angiogenic factors VEGF-A in stromal cells. Previous studies have highlighted the role of VEGF in the early angiogenic processes associated with postmenstrual regeneration of the endometrium [101–103]. The temporal and spatial distribution of VEGF is essential for the rapid burst of angiogenesis that occurs at postmenstrual repair [101].

The endometrial stroma secretome acts in an autocrine and paracrine manner to facilitate decidual differentiation, maternal angiogenesis thus influencing trophoblast invasion and placentation [19, 23]. We show here aberrant PlGF primed EnSCs secretome (CM) by displaying low levels of secreted VEGF-A despite the increase in HIF-1α and cellular VEGF-A levels. The decrease in secreted protein levels of VEGF-A was a puzzling result, however it is thought that intracellularly activated VEGF-A (in EnSCs) can likely interact with the receptors located within the cell exhibiting an intracrine signaling, causing a decline in secretion levels of VEGF-A [104, 105]. Another possible mechanism could be the activation of autocrine signaling in EnSCs, where secreted VEGF-A follows a negative feedback loop binding to its extracellular receptors causing a decrease in the bioavailability of VEGF-A in the CM [106, 107]. Lastly, secreted PlGF upon HIF-1α activation in EnSCs could act as an antagonist to its structurally related analog VEGF-A. HIF-1α transcriptional activity is known to be a key regulator of PlGF secretion [108, 109]. In line with this, HIF-1α -PlGF activation could be conserved as bidirectional regulation in EnSCs. Therefore, the secreted PlGF could act as an antagonist for VEGF-A present in CM by formation of biologically inactive PlGF/VEGF heterodimers [110]. These proposed mechanisms are yet to be fully characterized.

During endometrial sprouting angiogenesis, growth factors and cytokines from the stroma and surrounding uterine microenvironment activate the quiescent endothelial cells lining the vasculature, to degrade the extracellular matrix and invade the surrounding tissue to form new capillaries [19, 20, 23]. Here, we aimed to better elucidate the effects of PlGF-induced secreted factors on HUVECs by verifying its effects on angiogenic sprouting behaviour in HUVECs. VEGF-A is known to be mitogenic for endothelial cells [111]. The binding of VEGF-A to its receptors on endothelial cells triggers a series of intracellular signaling events, that mediate cellular responses aiding in proliferation, migration and vascular permeability [111, 112]. The VEGF-A pathway is tightly regulated and balanced in normal physiological conditions, but dysregulation of this pathway can contribute to various diseases, including cancer and vascular disorders [113, 114]. VEGF-A displays a concentration-dependent activity to induce endothelial cell proliferation, thus facilitating sprouting and anastomosis *via* a VEGF/Notch-dependent mechanism [84, 115]. In our study, PlGF-CM rendered a negative angiogenic modulation in HUVECs with impediment in proliferation, migration and pathological hypersprouting. VEGF-A and Notch signaling pathways often interact in a coordinated manner to fine-tune angiogenesis and vascular development [84, 116]. Low gene expression of notch effectors in PlGF-CM treated HUVECs, confirms poor notch regulation owing to low secreted VEGF-A by EnSCs. VEGF-A activates Dll4 expression in the tip cell by binding to VEGF receptors [115]. This receptor binding activates the Notch signaling pathway in the stalk cell leading to a suppression of the tip cell phenotype. Low levels of notch ligand (Dll4) and upregulation of VEGFR2 protein explains the hypersprouting behaviour observed in PlGF-CM treated HUVECs, causing failure in establishing proper balance between tip and stalk cells. Deranged Notch regulation between endothelial cells also resulted in downregulation of VEGFR1 and VEGF-A protein levels in HUVECs. In line with the reported findings, decreased expression of Notch and VEGFR1 was found in endothelial cells of decidua associated with early pregnancy loss [117]. The above data validates the crosstalk between the stroma and endothelial compartments aiding in cell-cell communication to regulate endometrial angiogenic function.

The proteomic characterization of CM secreted by EnSCs upon PlGF stimulation displayed upregulation of many ECM associated biomolecules. These molecules are also known to impinge angiogenesis by directly affecting endothelial cell phenotypes and functions [118]. Type I collagen is reported to play a major role in endothelial cell morphogenesis involving suppression of cAMP-dependent protein kinase A and induction of actin polymerization [119]. This finding is in keeping with our recent study, where we observed the PlGF-induced increase in both Type1 collagen and cell stiffness via increased actin polymerization [40]. Matrix metalloproteases (MMPs) are well known inflammatory mediators controlling endothelial proliferation and survival in pathological vessel modifications [120]. Using bioinformatic GO enrichment analysis, we show activation of pathways associated with pathological angiogenesis (atherosclerosis and hypertrophic cardiomyopathy) in the stromal secretome mediated by aberrant PlGF. The appearance of acute atherosis was found in the walls of spiral arteries of uteroplacental circulation in some PE cases [121, 122]. Acute atherosis lesions reportedly correlate with early stages of atherosclerosis [122]. Thus, we postulate the change in the composition of the ECM associated proteins together with angiogenic imbalances in PlGF-CM generates a pathological *inflammatory-like* microenvironment that modifies the angiogenic sprouting behaviour in HUVECs.

Establishment of a low-resistance vascular system is essential for adequate spiral artery remodelling and normal placentation [123]. In humans, it is estimated that between 50 and 100 spiral arteries are required to be transformed to support a healthy pregnancy [86, 124]. The importance of sufficient uterine vessel adaption is critical because by term (37 weeks) perfusion of the uterus increases from < 1% of cardiac output to 25%, of which 90% is carried through the limited number of physiologically transformed spiral arterioles into the intervillous space of the placenta [2, 123, 125]. Endothelial barrier function maintains vascular integrity by balancing vascular permeability and resistance [126]. We observed that low VEGF-A and high ECM associated biomolecules in PlGF-CM impaired barrier function in HUVECs (high cell impedance), resulting in poor permeability and increased vascular stiffness. This increase of vascular resistance and stiffness at the junctional interface of HUVECs impeded BeWo cell invasion. Therefore, high levels of endometrial PlGF could form high-resistant vessels in the endometrium resulting in insufficient trophoblast invasion as manifested in PE placentas.

SGK1 is recognized as a critical endometrial regulator participating in both uterine receptivity and pregnancy [55]. It is known to play a mechanistic role in maintaining the functional reproductive axis [55, 127]. However, to date, the exact role of SGK1 in endometrial vessel development remains unclear. Various studies report the critical role of SGK1 in regulating inflammation in vascular diseases. Xi et al., showed first evidence for a role of SGK1 in hypoxia mediated pulmonary hypertension by inducing pro-inflammatory reaction. Lack of SGK1 attenuated hypoxia induced pro-inflammation response and improved arterial remodelling [128]. Similarly, Baban et al., reported that activation of SGK1 signaling improved inflammation-mediated pro survival pathways, causing adverse cardiac remodelling in ischemic- reperfusion injury [129]. We report that PlGF treatment with SGK1 inhibition in EnSCs attenuated hypoxia and increased the secreted levels of VEGF-A. In addition, the corresponding CM improved endothelial migration, normal tube formation ability and vascular permeability as seen in Con-CM. Our study also cements the role of SGK1 as a key molecule, mediating PlGF induced angiogenic pathway by promoting hypoxia and differentially regulating angiogenic cues for pathological vessel development. HIF-1*α* overexpression is reported to be expressed in the PE placentas and known to regulate the production of sFlt-1 and soluble endoglin (sEng), causing the angiogenic imbalance [130, 131]. Furthermore, HIF-1*α* overexpression reportedly induces a Hemolysis, Elevated Liver enzymes and Low Platelets (HELLP) syndrome-like phenotype and fetal growth restriction in pregnant mice [132]. These data help us to postulate the plausible role of dysregulated endometrial SGK1 in enhancing hypoxia during uterine vascularization and placentation as presented in PE. Also, our findings highlight the potential for selective inhibition of SGK1 in stroma to reverse the pathological switch activated by aberrant PlGF. This study uncovers a new role of SGK1 in PE pathogenesis and identifies SGK1 as an attractive therapeutic target.

**Fig. 7:**
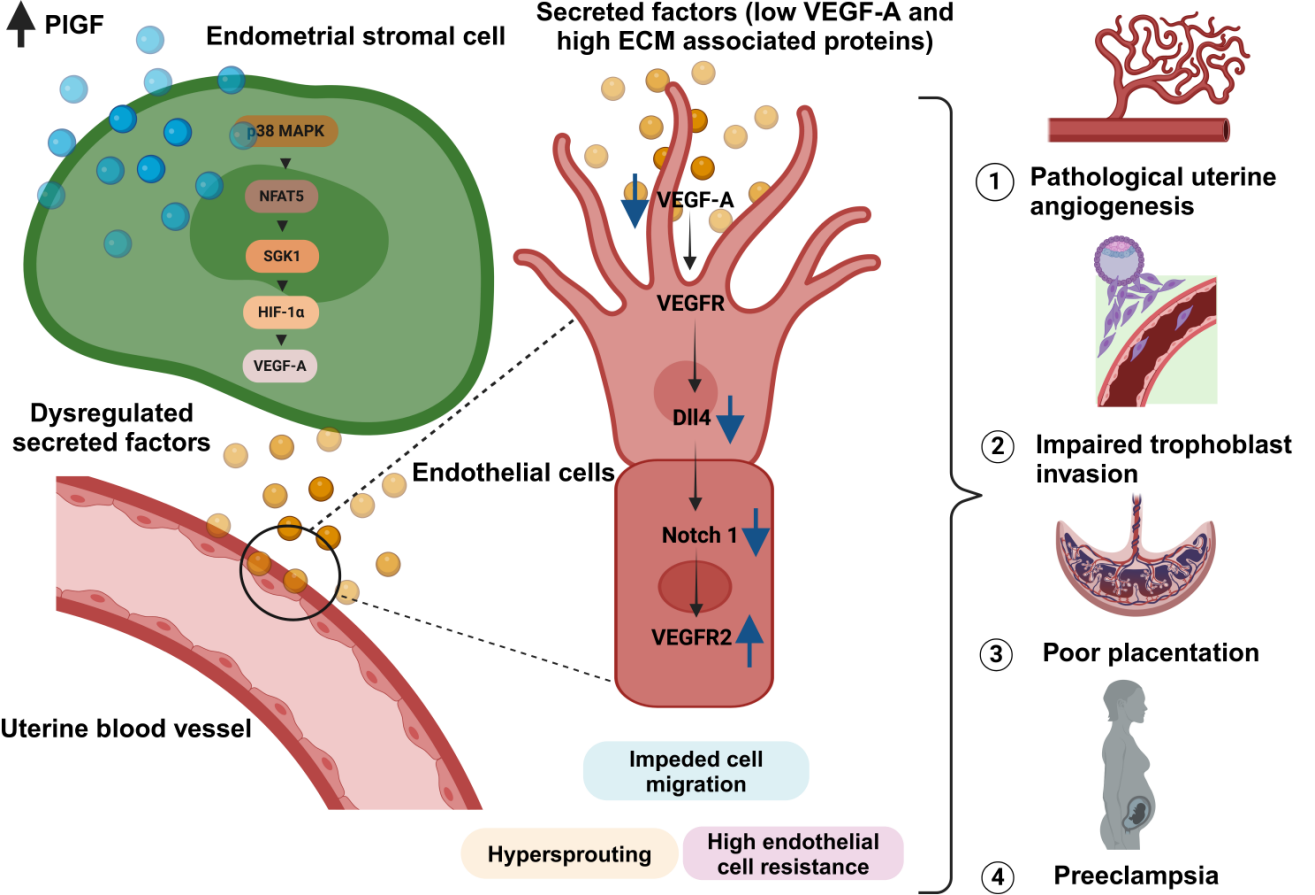
Graphical abstract describing the effect of pathological PlGF levels in altered uterine endometrial angiogenesis and its plausible role in PE pathology. Aberrant levels of endometrial PlGF activates NFAT5-SGK1-VEGF-A signaling axis in uterine stromal cells. Activation of this signaling cascade presents negative angiogenic cues to endothelial cells, with deregulated secreted protein cargo (decreased angiogenic factor VEGF-A and increased ECM associated proteins). PlGF mediated secreted factors supports abnormal vessel development in HUVECs, with dysregulation of Notch-VEGF signaling. Aberrant PlGF triggered stromal-endothelial paracrine signaling results in hypersprouting, high cellular resistance and impaired BeWo invasion through HUEVCs. Hypersprouting and high cellular impedance in HUVECs confirm pathological uterine vascularization upon deregulated endometrial PlGF. Thus, we postulate such aberrant uterine angiogenesis prior to pregnancy will likely lead to poor quality maternal vessels, inadequate trophoblast invasion causing poor placentation as seen in PE pregnancy (Images created with BioRender)

Collectively, our findings support the conjecture that dysregulated endometrial PlGF could switch between the controlled physiological angiogenesis by disrupting the uterine stromal – endothelial paracrine mechanism, resulting in poor quality spiral artery modification thereby impeding trophoblast invasion and thus development of PE (Fig. 7). In keeping with this, Doppler studies showed a higher uterine artery pulsatility index during early gestation (weeks 11–13) and could identify at least 50% of patients who subsequently developed PE [133]. Interestingly, additional Doppler ultrasound studies have shown that during the late secretory phase, endometriosis is linked with increased sub-endometrial blood flow [134]. The inverse correlation between the amount of perfusion before pregnancy and the probability of pregnancy complications may, although untested, be relevant to other disorders including abnormal uterine bleeding, polycystic ovary syndrome and unexplained infertility. Notwithstanding, whilst we shed light on a new pathway, other contributing factors such as inadequate decidualization, deregulated uNK cell function, impaired activation of trophoblast interaction, trophoblast cell death, reprogramming /epigenetic changes (DNA methylation or post translational histone tail modifications) or a combination of the above may lead to dysregulation of spiral artery transformation and PE [16, 135–139].

Cyclic endometrial remodelling and menstruation is a pre-requisite for the human uterus in preparation for future pregnancy [140]. Hence, we posit that local endometrial disorders i.e. poor-quality uterine vessels (prior to pregnancy) will result in an unwarranted ripple effect with the ‘crescendo’ or end result being reduced placental function as presented in PE. Our findings presented here are further supported by a recent study employing single-cell transcriptomics to survey distinct molecular faces of PE subtypes [141]. Systematic molecular characterization revealed imbalances hallmarking angiogenic and extracellular matrix dysfunction in placentas from early onset PE. Intriguingly, stromal cells and vasculature reflected an inflamed, stressed, anti-angiogenic environment only in early PE groups. Thus, we could speculate that PE is primarily a disease of impaired endometrial preconditioning, which likely confers protection against abnormal hyperinflammation.

The correlation between abnormal endometrial PlGF and the corresponding circulating levels during early pregnancy driving abnormal pregnancy outcome remains unclear. PlGF, exists in at least four isoforms due to alternative mRNA splicing of the PlGF primary transcript [142, 143]. The main distinction amongst the four isoforms are that PlGF-1 and − 3 are non-heparin binding and can (potentially) affect targets in a paracrine manner, whereas PlGF-2 and − 4 have additional heparin-binding domains and most likely work in an autocrine way [142, 143]. The major isoforms are thought to be PlGF-1 and PlGF-2 and are thought to have different functions [143]. The addition of PlGF-1 to a spontaneously transformed first-trimester cytotrophoblast cell line stimulated cell proliferation whilst PlGF-2 had little effect [144]. In contrast, the addition of PlGF-1 had little effect on endothelial cell proliferation while this was inhibited by PlGF-2 [144]. Therefore, more careful analysis is required to delineate which isoform in the endometrium contributes to the pathogenesis of this disease. Our reanalysis of single-cell RNA sequencing of the human endometrium reveals that PlGF is expressed by various endometrial cell types (data not shown) with the highest expression levels observed in the stromal cells, endothelial cells and mesenchymal stem cells (MSCs) [145]. Intriguingly, the conditioned medium of PE placental MSCs impair trophoblast invasion and angiogenesis of endothelial cells, indicating a detrimental paracrine profile [146]. Furthermore, MSCs derived from the decidual component of PE placentas exhibited compromised function in response to oxidative stress and accelerated senescence compared with normotensive placentas [146–148]. In keeping with this, in our proteomic analysis we do see a modest upregulation of senesce marker β-galactosidase (data not shown). Taken together, we posit that aberrant expression of endometrial PlGF on MSCs could additionally deregulate the normal cellular activity, potentially leading to premature cellular senescence causing placental aging as seen in obstetric complications such as PE, IUGR and still birth [149, 150] and further work is required to validate this hypothesis.

Literature shows strong evidence on high levels of circulating PlGF in individuals with various diseases such as cancer (breast, melanoma, leukemia), auto-immune diseases (rheumatoid arthritis, Systemic Lupus Erythematosus), coronary heart disease, and neovascular age-related macular degeneration. Whether PlGF contributes to or is a result of these diseases remains to be determined [28, 29]. Furthermore, the knowledge on genetic determinants of circulating pathological levels of PlGF is limited. A recent study by Ruggerio et al., conducted the first genome-wide association study, to identify genetic variants that explain alterations in circulating PlGF concentrations [28]. The plausible candidate genes identified to be associated with PlGF circulating levels were *NRP1, FLT1 and RAPGEF5* [28]. Intriguingly these molecules have been implicated with different PE models [151–153]. Another study by Vodolazkaia et al., reported that genetic variants in *PlGF* rs2268613 gene may increase the PlGF plasma levels [154]. These findings thus provide new candidate genes and new insights into mechanisms by which PlGF is regulated in physiological and pathophysiological states.

PE is a complex condition involving multiple systems and various contributing factors [9]. Its development results from a combination of immunological, genetic and environmental influences, leading to systemic maternal endothelial dysfunction and impaired placental function [155]. Significant (high) risk factors include a history of PE, chronic hypertension, pre-existing diabetes mellitus, chronic kidney disease history and autoimmune conditions like antiphospholipid syndrome (APS) [155]. Additional risk (moderate) factors encompass advanced maternal age (> 40 years), body mass index [BMI] ≥ 35 kg/m2, and the use of assisted reproductive technologies (ART/IVF) [155]. According to the NICE recommendations, the presence of one high-risk factor or more than one moderate risk factor is considered high risk for pre-eclampsia [13]. Interestingly, abnormal PlGF levels are also (independent of pregnancy) associated with some of these risk factors, such as diabetes, obesity and hypertension [156–159]. Interestingly, these conditions are also linked with increased SGK1 expression [160–163]. Taken together, these studies argue that certain genetic variations as previously stated or lifestyle factors may predispose women to abnormal PlGF levels before pregnancy, potentially augmenting the expression of the PlGF-NFAT5-SGK1 axis and promoting inadequate vessel quality in the endometrial microenvironment. Subsequently, during pregnancy, these compromised vessels may contribute to abnormal placentation, resulting in an imbalance in circulating angiogenic factors. In conclusion, our results shed light onto the new prospect and advances of PlGF-NFAT5-SGK1 signaling axis in endometrial stromal cells. While careful evaluation of the broad implications of PlGF is still necessary, this study identified both NFAT5 and SGK1 as promising targets for treatment strategies to improve vascularization prior to pregnancy and help improve adverse pregnancy outcome such as PE.

### Electronic supplementary material

Below is the link to the electronic supplementary material.

Supplementary Material 1

Supplementary Material 2

## Data Availability

The proteomic datasets supporting the results in this article are available in the PRoteomics IDEntification database (PRIDE) with the accession number PXD051697. The source data behind the other data available in the figures can be found in the supplementary data file 2.
