# Supplementary material for: Placental growth factor mediates pathological uterine angiogenesis by activating the NFAT5-SGK1 signaling axis in the endometrium: implications for preeclampsia development
Source: Biol Res. 2024 Aug 17;57:55. doi: 10.1186/s40659-024-00526-w (PMC11330076; doi:10.1186/s40659-024-00526-w)
Supplement: Supplementary file 1 — Supplementary Material 1 [file 40659_2024_526_MOESM1_ESM.pdf]

Supplementary Information

Placental growth factor mediates pathological uterine angiogenesis by activating the NFAT5-SGK1 signaling axis in the endometrium: Implications for preeclampsia development.

Supplementary 1

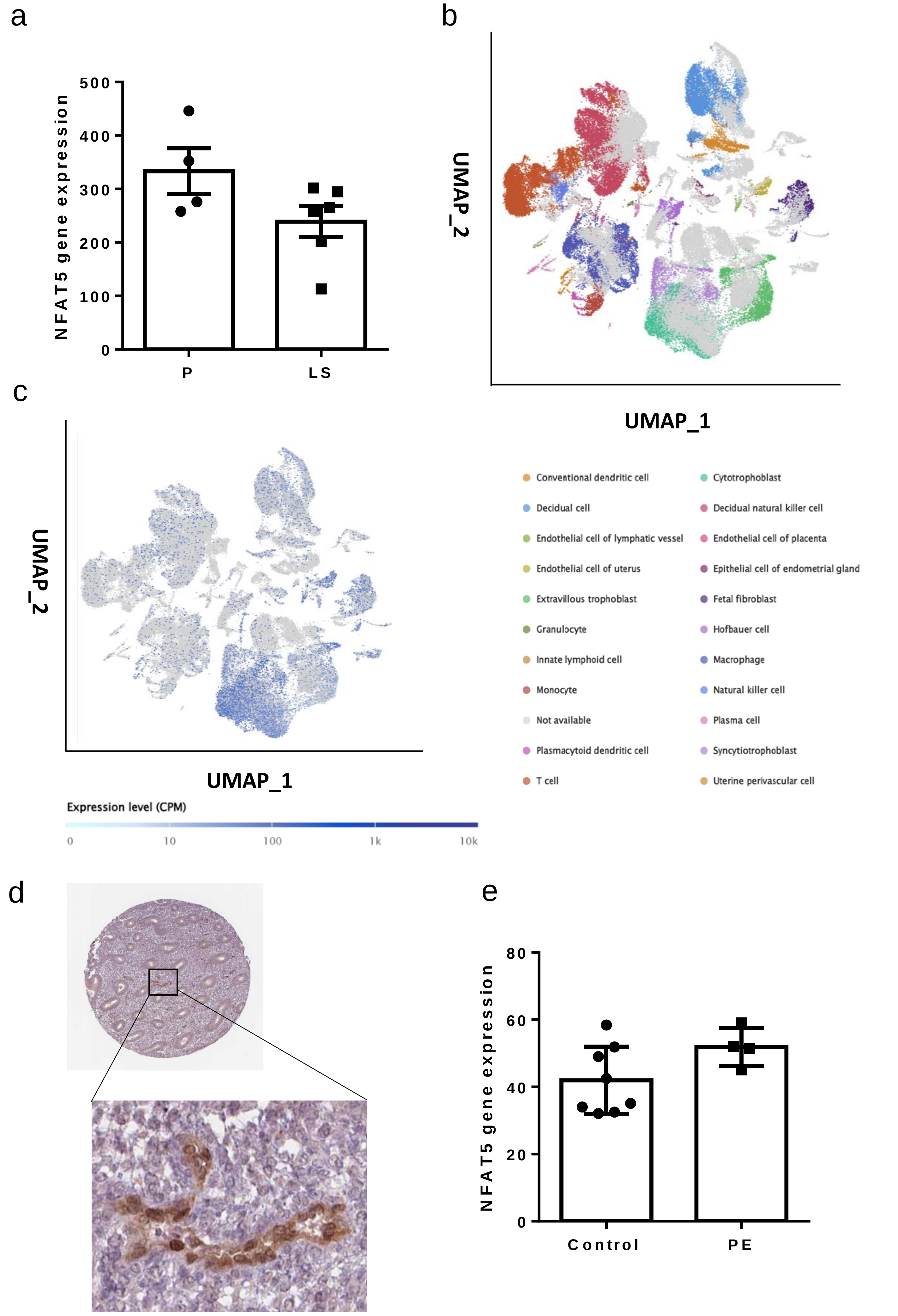

Supplementary Figure 1: a. NFAT5 gene expression value across the menstrual cycle in the proliferative (P) and late secretory (LS) phase (*GDS 2052*). b-c. UMAP projections of the dataset from Single-cell reconstruction of the early maternal-fetal interface in humans, b represents cell lineages of the decidua and placenta, c represents NFAT5 expression across the different decidual and placental cell population.d. NFAT5 protein expression on endometrial tissue samples analyzed from Human Protein Atlas. NAFT5 is expressed throughout the endometrium and staining was highest in the stroma near to the blood vessels e. NFAT5 gene expression value in the pre-symptamatic deciduas of PE patients compared with healthy (control) pregnant deciduas (*GDS 3467*).

Supplementary 2

a

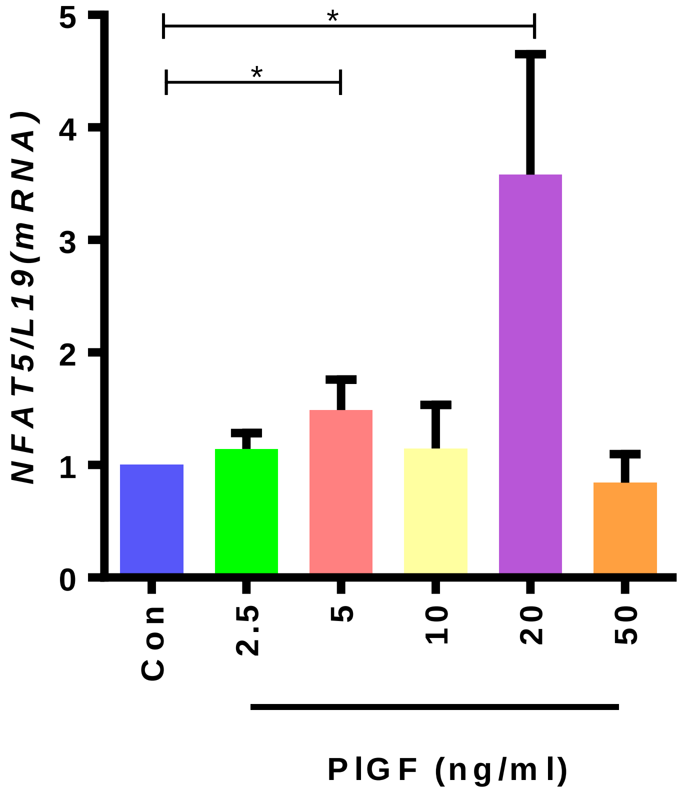

b

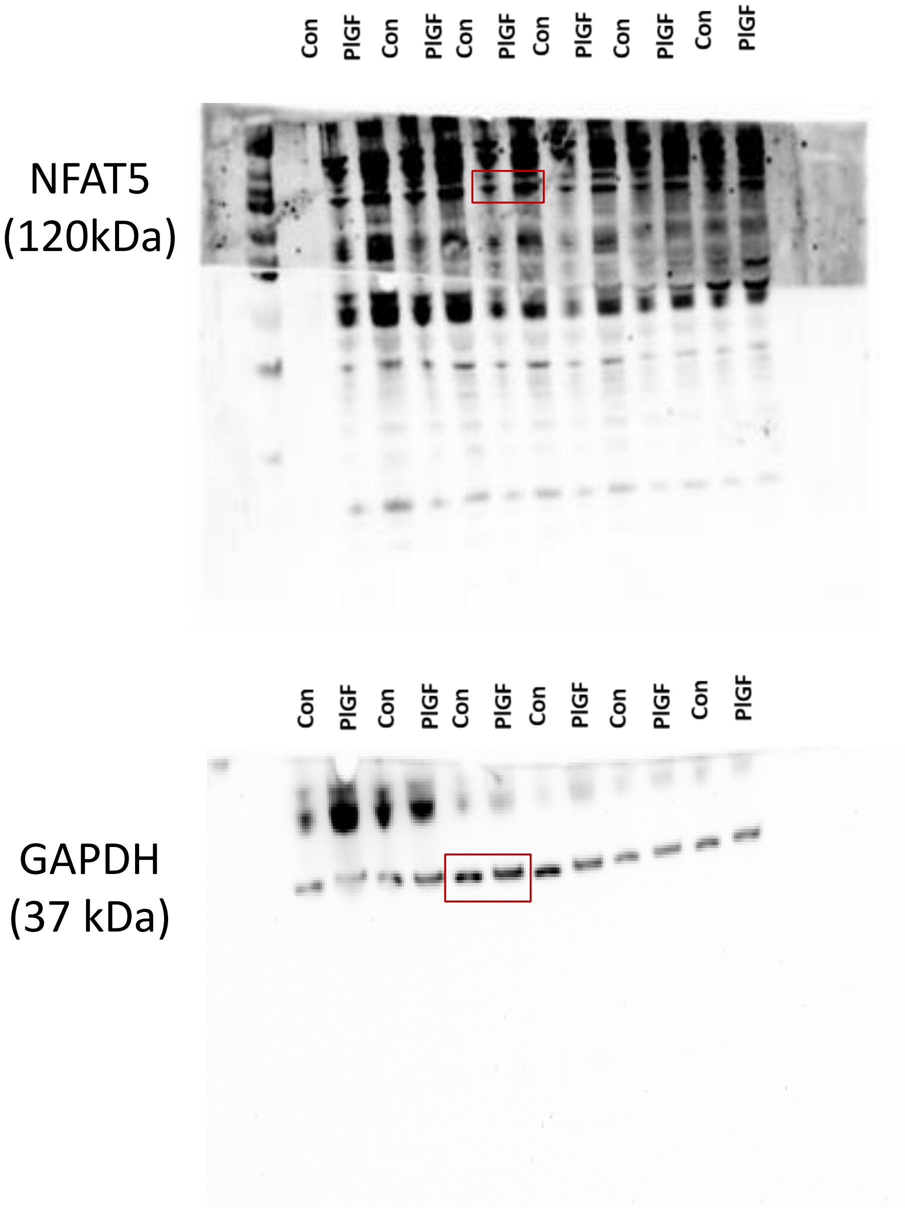

Supplementary Figure 2: a. qPCR determining NFAT5 expression in EnSCs treated with varying concentrations of PlGF (2.5 -50 ng/ml) for 24 hours. (n=4, *, $p < 0.05$) b. Original western blot membrane of blots represented in figure 1b.

Supplementary 3

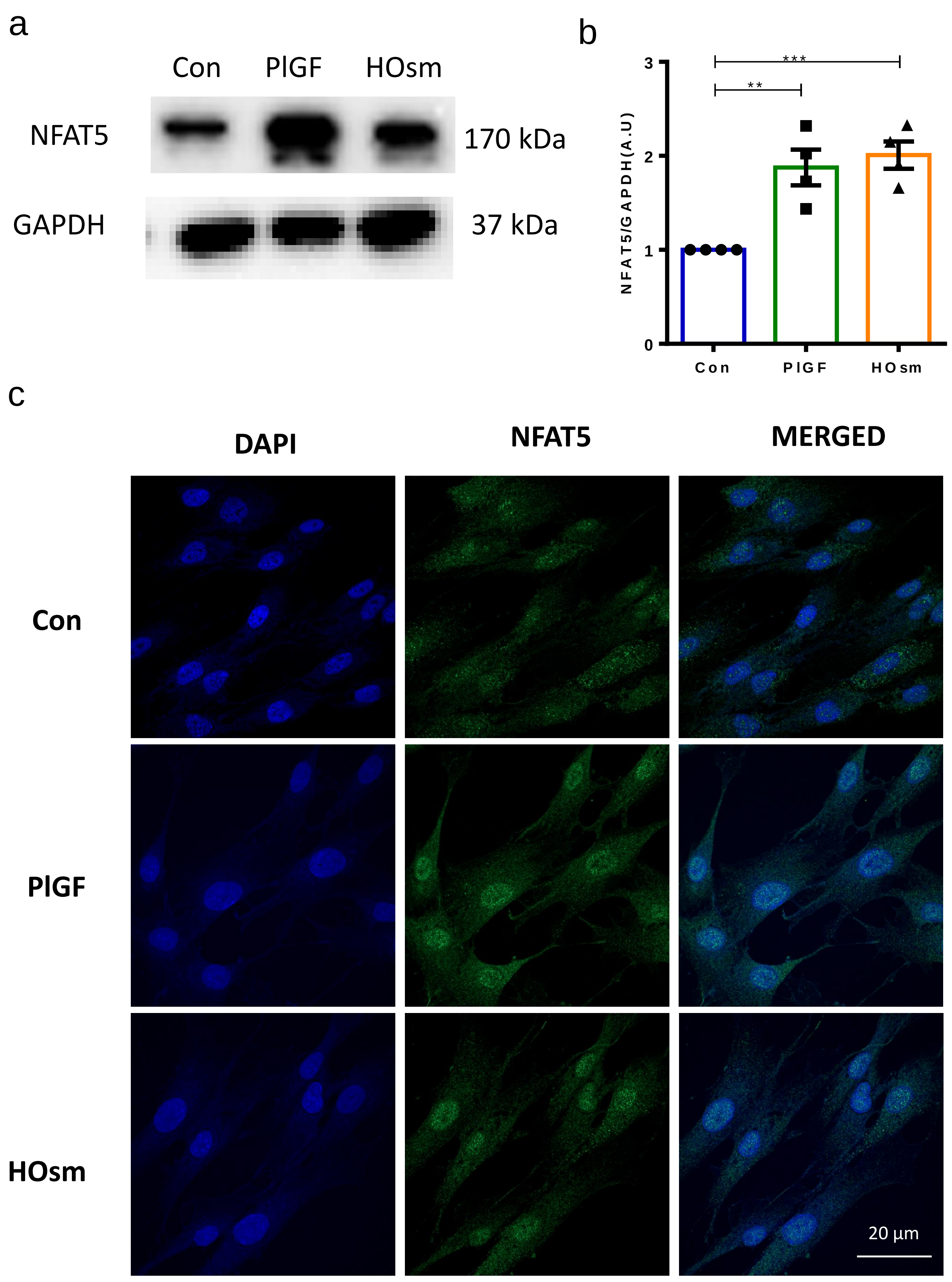

Supplementary 3: a. Original Western blot analysis of NFAT5 protein with GAPDH as loading control in untreated (Con), PlGF and HOsm treated EnSCs. b) Average NFAT5 protein levels after 6 days treatment with PlGF and 3 h treatment with Hosm (n=4, **, p 0.01, ***, p < 0.001). The samples are represented after normalization with untreated control (Con) (n=3). c) Immunofluorescence images confirms nuclear translocation of NFAT5 from the cytosol when activated by HOsm (n=3). Scale bar: 20 µm. Data represented as arithmetic mean ± SEM. Significance was determined using student's unpaired two-tailed t-test with Welch's correction method.

Supplementary 4

p-p38 MAPK (43 kDa)

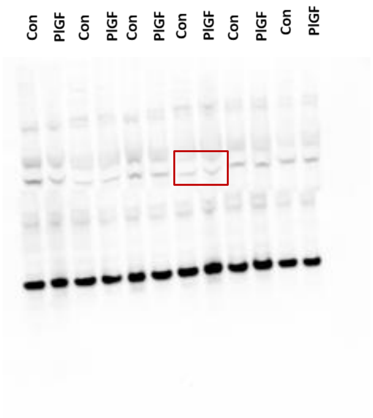

GAPDH (37 kDa)

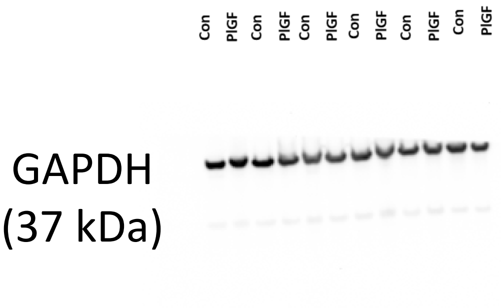

SGK1 (45-50 kDa)

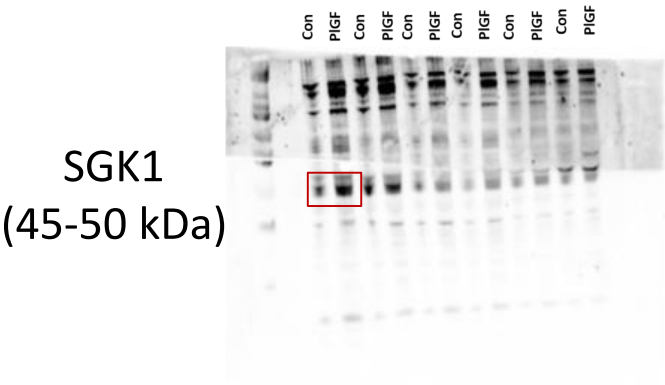

GAPDH (37 kDa)

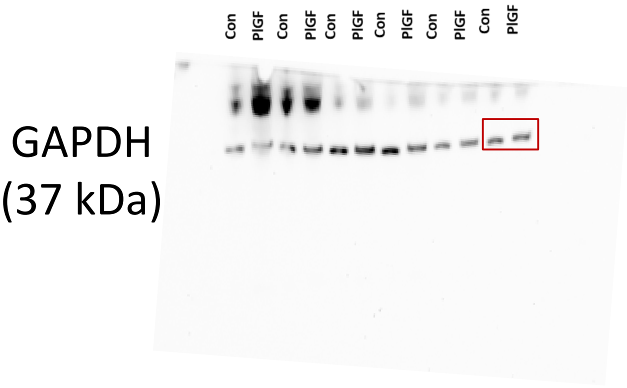

p-SGK1 (54 kDa)

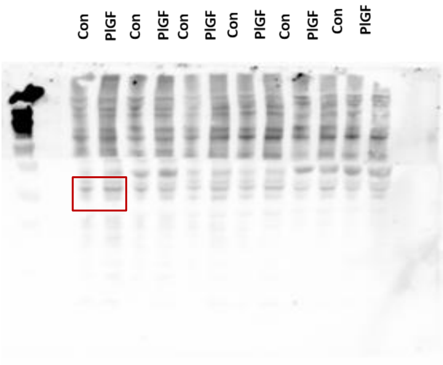

VEGF-A (27 kDa)

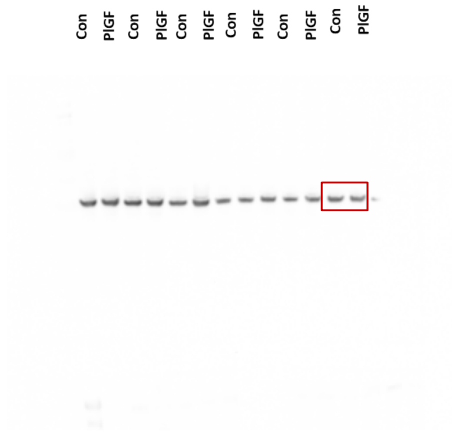

p38 MAPK (40 kDa)

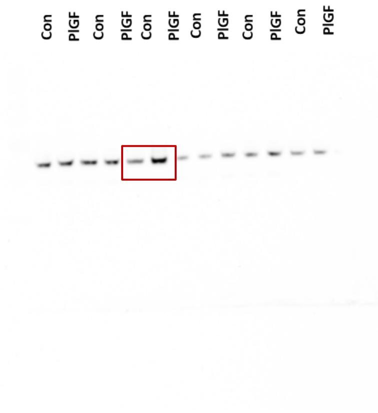

GAPDH (37 kDa)

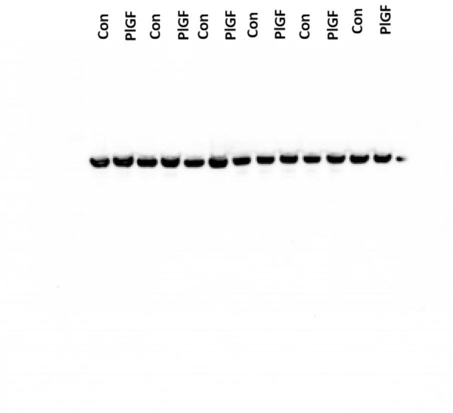

Supplementary Figure 4 : Original western blot membrane of blots represented in figure 2a.

Supplementary 5

a

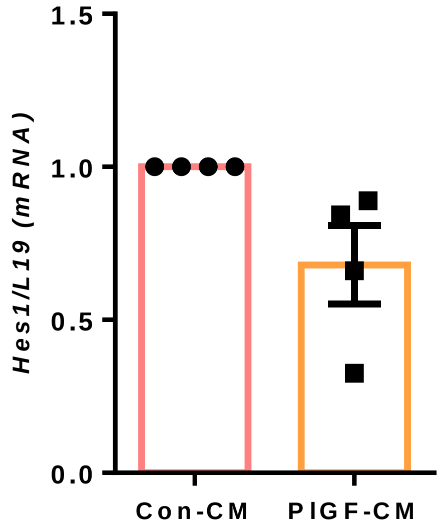

b

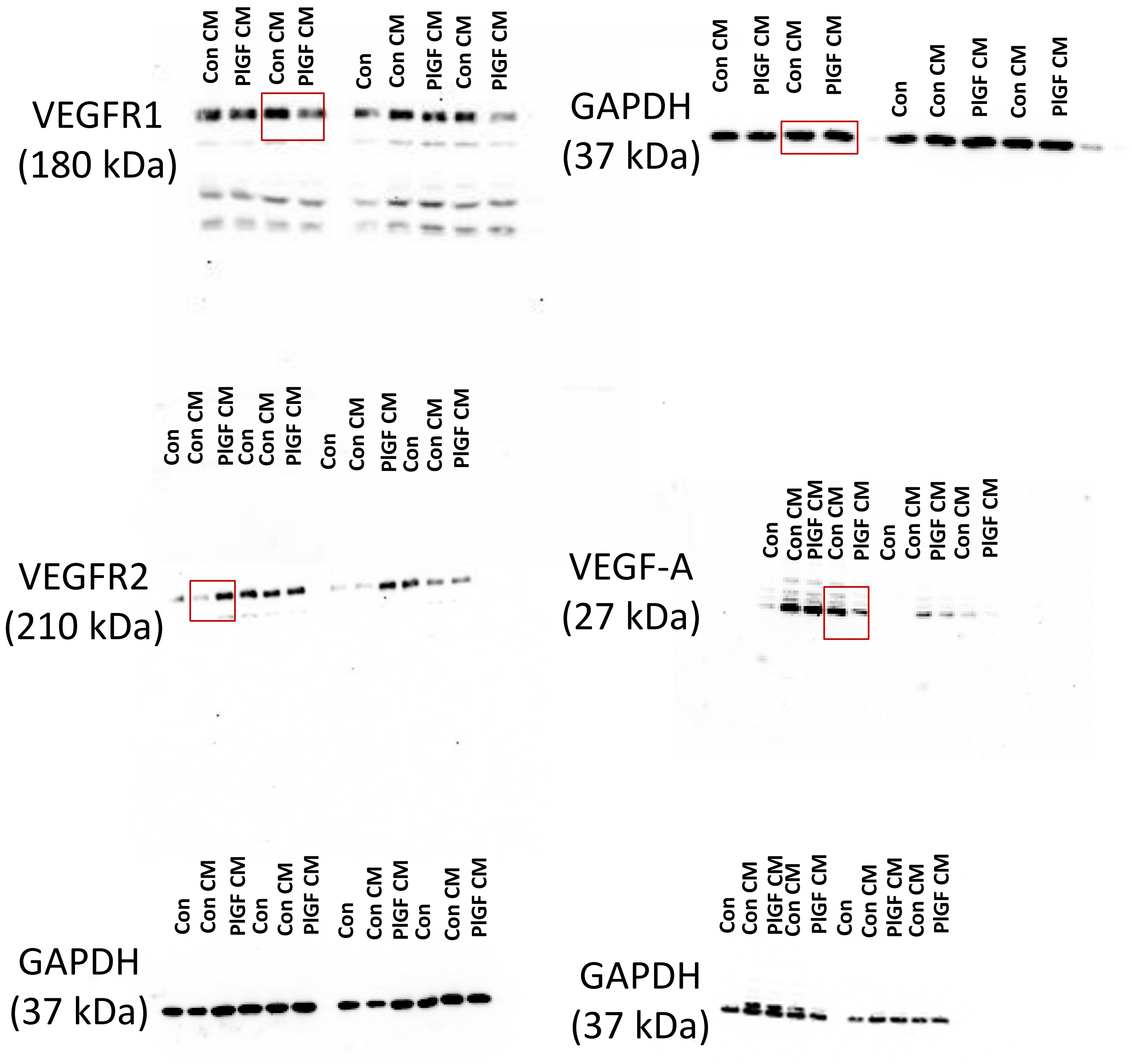

Supplementary Figure 8 : a. . qPCR analysis of Notch target gene (*Hes 1*) in Con-CM and PlGF-CM treated HUVECs. *L19* was used as a housekeeping control. (n=4). b. Original western blot membrane of blots represented in figure 4m.

Supplementary 6

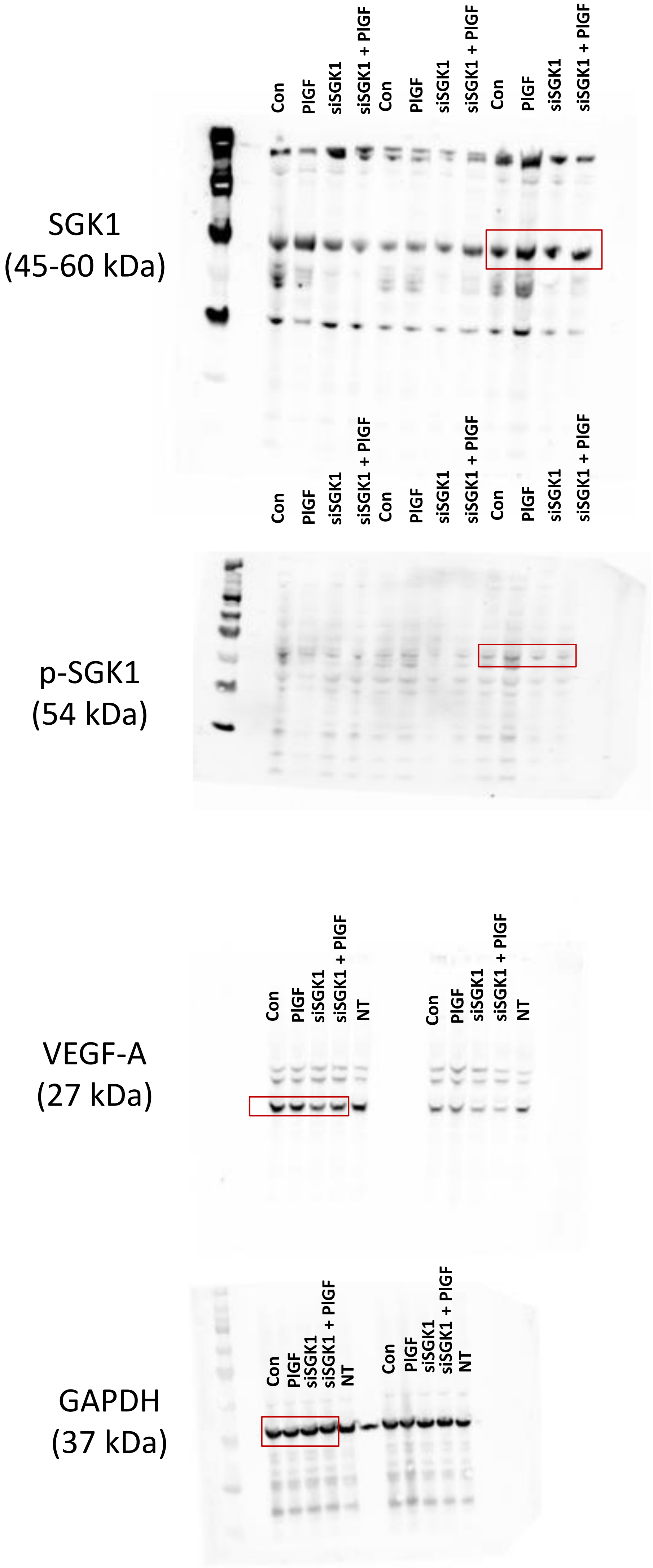

Supplementary Figure 8 : Original western blot membrane of blots represented in figure 5a.

Supplementary 7

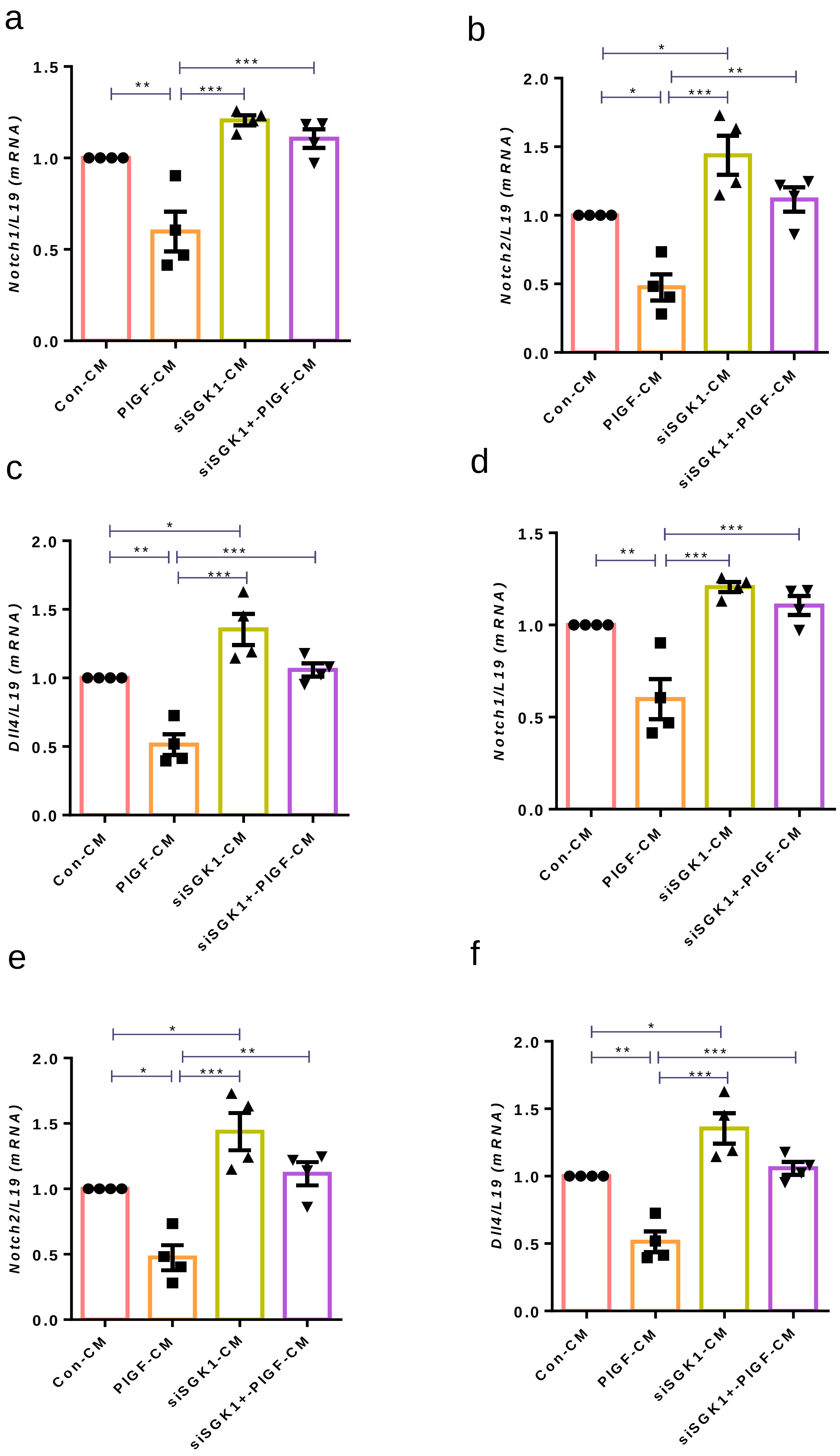

Supplementary Figure 7 : a-f. qPCR analysis of Notch receptors (*Notch1 and Notch2*), ligands (*Dll4 and Jagged-1*) and target genes (*Hey1and Hes1*) in Con-CM and PlGF-CM treated HUVECs. *L19* was used as a housekeeping control. (n=4, *, p<0.05, **, p < 0.01).

Supplementary 8

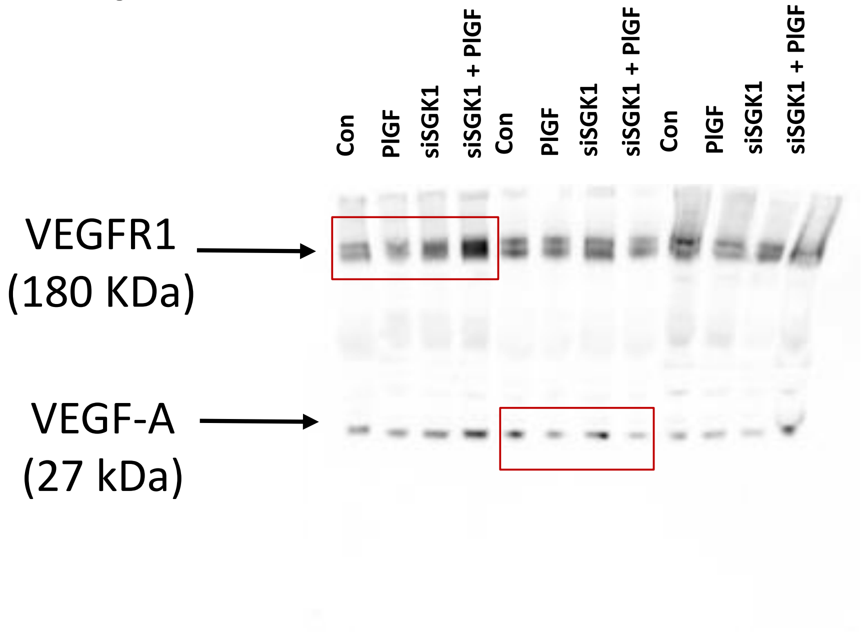

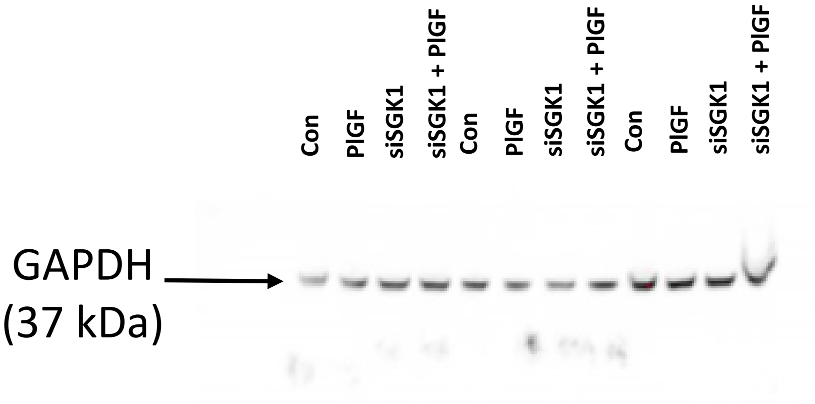

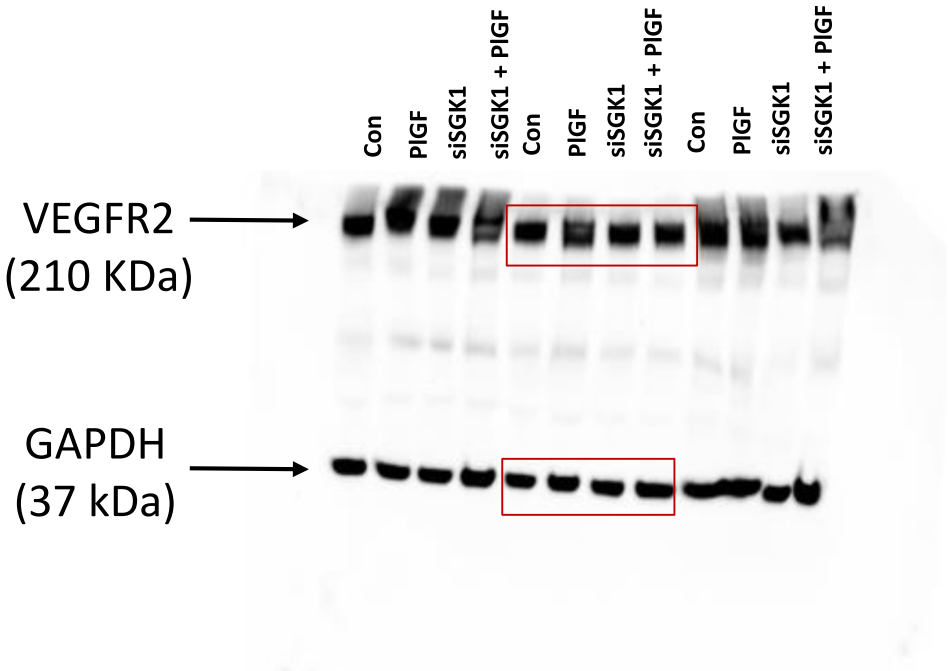

Supplementary Figure 8 : Original western blot membrane of blots represented in figure 6f.
